# Re-creation of a Key Step in the Evolutionary Switch from C_3_ to C_4_ Leaf Anatomy

**DOI:** 10.1016/j.cub.2017.09.040

**Published:** 2017-11-06

**Authors:** Peng Wang, Roxana Khoshravesh, Shanta Karki, Ronald Tapia, C. Paolo Balahadia, Anindya Bandyopadhyay, W. Paul Quick, Robert Furbank, Tammy L. Sage, Jane A. Langdale

**Affiliations:** 1 Department of Plant Sciences, University of Oxford, South Parks Road, Oxford OX1 3RB, UK; 2 Department of Ecology and Evolutionary Biology, University of Toronto, Toronto, ON M5S3B2, Canada; 3 International Rice Research Institute (IRRI), Los Banos 4030, Laguna, the Philippines; 4 Department of Animal and Plant Sciences, University of Sheffield, Sheffield S10 2TN, UK; 5 CSIRO, Canberra, ACT 2601, Australia; 6 ARC Centre of Excellence for Translational Photosynthesis, Research School of Biology, Australian National University, Acton, ACT 2601, Australia

**Keywords:** C_4_ rice, GLK transcription factors, Kranz anatomy, evolution, chloroplasts, organelle development, intracellular signaling, plasmodesmata

## Abstract

The C_4_ photosynthetic pathway accounts for ∼25% of primary productivity on the planet despite being used by only 3% of species. Because C_4_ plants are higher yielding than C_3_ plants, efforts are underway to introduce the C_4_ pathway into the C_3_ crop rice. This is an ambitious endeavor; however, the C_4_ pathway evolved from C_3_ on multiple independent occasions over the last 30 million years, and steps along the trajectory are evident in extant species. One approach toward engineering C_4_ rice is to recapitulate this trajectory, one of the first steps of which was a change in leaf anatomy. The transition from C_3_ to so-called “proto-Kranz” anatomy requires an increase in organelle volume in sheath cells surrounding leaf veins. Here we induced chloroplast and mitochondrial development in rice vascular sheath cells through constitutive expression of maize *GOLDEN2-LIKE* genes. Increased organelle volume was accompanied by the accumulation of photosynthetic enzymes and by increased intercellular connections. This suite of traits reflects that seen in “proto-Kranz” species, and, as such, a key step toward engineering C_4_ rice has been achieved.

## Highlights

• Organelle development in rice vascular sheath cells is induced by maize *GLK* genes
• Cells with activated organelles form more plasmodesmatal connections than wild-type
• *GLK*-induced anatomy mimics a key step in the evolutionary transition from C_3_ to C_4_
• The intermediate proto-Kranz leaf anatomy appears to bear no fitness cost in rice

## Teaser

Wang et al. report that constitutive expression of maize *GOLDEN2-like* genes induces a suite of traits in C_3_ rice leaves that is reminiscent of proto-Kranz anatomy, an intermediate state in the evolutionary trajectory from C_3_ to C_4_. A key step toward engineering C_4_ rice has thus been achieved.

## Introduction

The C_4_ photosynthetic pathway evolved over 60 times independently in a diverse range of flowering plant species, with trajectories from C_3_ to C_4_ apparent in a number of lineages [1]. In most C_4_ plants, photosynthetic reactions are compartmentalized between two cell types that are arranged in concentric wreaths around closely spaced veins. This cellular arrangement, which is referred to as Kranz anatomy [2, 3], facilitates initial fixation of carbon dioxide in the outer mesophyll cells followed by decarboxylation and refixation by ribulose bisphosphate carboxylase/oxygenase (RuBisCo) in the CO_2_-enriched environment of the inner sheath cells (reviewed in [4]). In most C_4_ species, the bundle sheath cells surrounding the vein are the site of refixation, but in some grasses an inner layer of mestome sheath cells with thick suberized cell walls play this role (either instead of [5] or in addition to [6, 7] the bundle sheath cells). A critical step in the evolution of C_4_ was thus the functionalization of bundle sheath and/or mestome sheath cells for photosynthesis (reviewed in [8, 9]).

A long-term project is underway to introduce the C_4_ pathway into the C_3_ crop rice, with predictions of up to a 50% yield increase if successful [10, 11] (https://c4rice.com). One of the major challenges in this regard is the need to introduce Kranz anatomy into the rice leaf. Morphological analyses of extant species suggest that Kranz evolved in a stepwise fashion, with “proto-Kranz”- and “C_2_”- type anatomies representing intermediate steps along the C_3_-to-C_4_ trajectory [9]. In both monocots [12] and eudicots [13, 14, 15, 16], proto-Kranz anatomy is characterized by increased organelle volume in the bundle sheath and/or mestome sheath cells around the leaf vasculature (collectively referred to as vascular sheath cells), with chloroplasts accumulating the photosynthetic enzyme RuBisCo and mitochondria accumulating the photorespiratory enzyme glycine decarboxylase. In the grasses, proto-Kranz species exist in the “PACMAD” clade, in which 24 independent origins of C_4_ have been identified [1, 12, 17, 18]. By contrast, there are no proto-Kranz or C_4_ species in the sister clade, to which rice belongs. As such, a rational first step toward engineering C_4_ rice is to induce proto-Kranz by activating chloroplast and mitochondrial biogenesis in vascular sheath cells.

GOLDEN2-like (GLK) transcription factors regulate chloroplast development in all land plant species examined [19, 20, 21, 22, 23, 24, 25], and ectopic expression of *OsGLK1* activates chloroplast biogenesis in rice vascular sheath cells [26]. However, enhanced chloroplast development in overexpression lines is not sustained beyond the seedling stage [26], suggesting that ectopic *OsGLK1* activity is suppressed by one of the post-transcriptional mechanisms that balance GLK-induced chloroplast development with leaf senescence [27, 28, 29]. On the basis of these findings, we postulated that sustained chloroplast development in the vascular sheath cells of rice might be achieved through the introduction of one of the two heterologous maize *GLK* genes (either *ZmG2* or *ZmGLK1* [20]). Here we validate that hypothesis and further demonstrate that enhanced chloroplast development leads to enhanced mitochondrial biogenesis, with both types of organelle accumulating metabolic enzymes. A proto-Kranz rice line has thus been generated.

## Results

### Constitutive Expression of *ZmG2* or *ZmGLK1* in Rice Induces Greening of Callus and Roots

**Figure 1:**
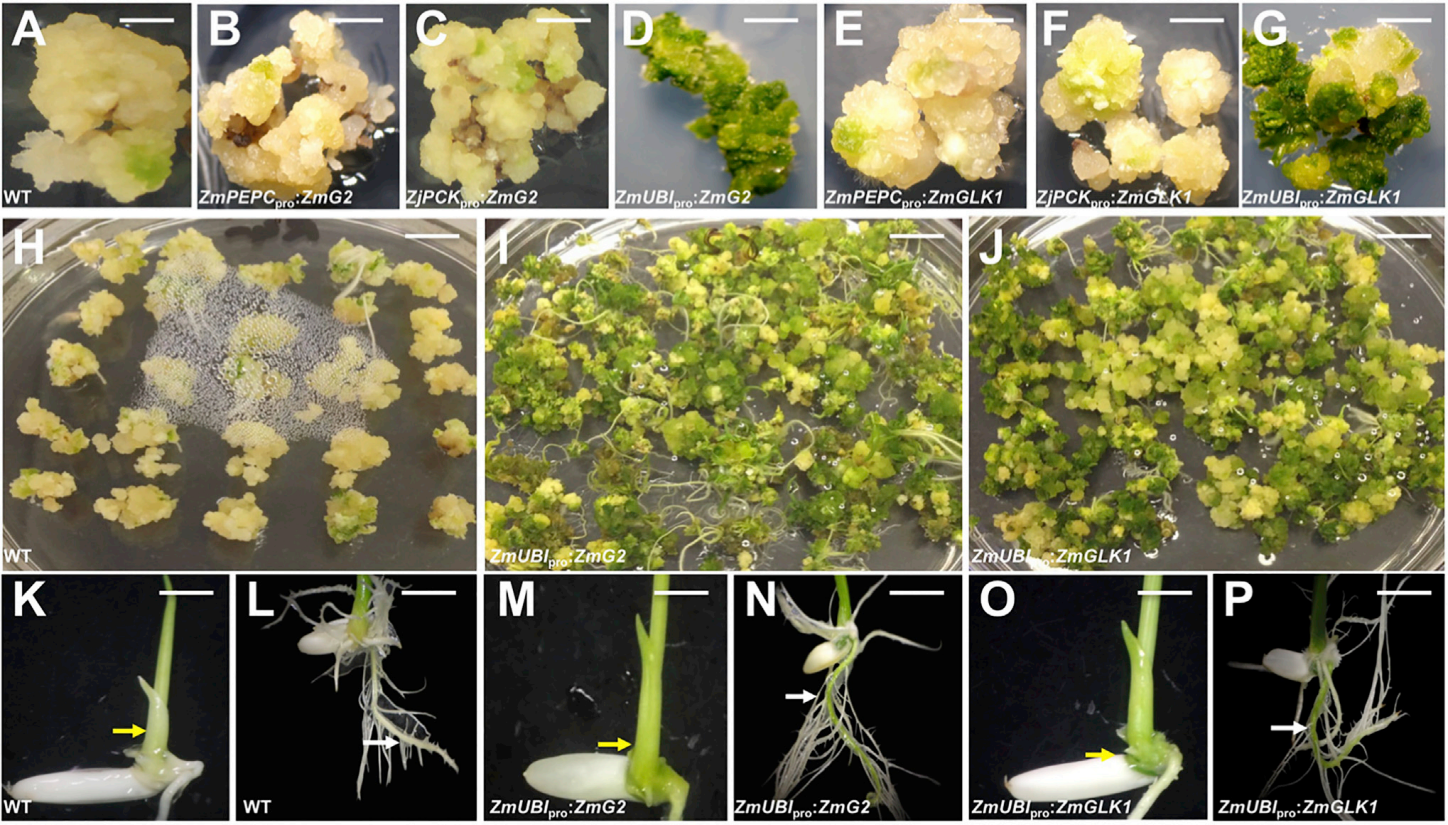
Constitutive Expression of *ZmG2* or *ZmGLK1* Leads to Enhanced Greening of Regenerating Callus and T1 Seedlings (A–G) Representative regenerating callus of T0 cultivar IR64 lines: wild-type (WT) (A), *ZmPEPC*_pro_:*ZmG2* (B), *ZjPCK*_pro_:*ZmG2* (C), *ZmUBI*_pro_:*ZmG2* (D), *ZmPEPC*_pro_:*ZmGLK1* (E), *ZjPCK*_pro_:*ZmGLK1* (F), and *ZmUBI*_pro_:*ZmGLK1* (G). (H–J) Representative regenerating callus of T0 cultivar Kitaake lines on cytokinin-containing media: WT (H), *ZmUBI*_pro_:*ZmG2* (I), and *ZmUBI*_pro_:*ZmGLK1* (J). (K–P) Representative T1 seedling shoots (K, M, and O) and roots (L, N, and P) of IR64 lines: WT (K and L), *ZmUBI*_pro_:*ZmG2* (M and N), and *ZmUBI*_pro_:*ZmGLK1* (O and P). Yellow arrows indicate hypocotyl, white arrows indicate primary root. Scale bars, 0.5 cm (A–G), 0.8 cm (H–J, L, N, and P), and 0.2 cm (K, M, and O). See also Figure S1 and Table S1.

To determine whether maize *GLK* gene function is sufficient to promote enhanced chloroplast development in rice, *ZmG2* or *ZmGLK1* transgenes were introduced under the control of constitutive or cell type preferential promoters. The maize *UBIQUITIN* promoter (*ZmUBI*_pro_) was used to drive constitutive expression [30], the *Zoysia japonica PHOSPHOENOLPYRUVATE CARBOXYKINASE* promoter (*ZjPCK*_pro_) for preferential expression in bundle sheath cells [31], and the maize *PHOSPHOENOLPYRUVATE CARBOXYLASE* promoter (*ZmPEPC*_pro_) for preferential expression in mesophyll cells [32]. Constructs were transformed into both *Oryza sativa spp. indica* cultivar IR64 and *spp. japonica* cultivar Kitaake (Figure S1; Table S1). A phenotypic consequence of constitutive expression was immediately apparent in that transgenic calli were green, whereas wild-type calli and those transformed with cell type preferential promoter constructs remained yellow (Figures 1A–1G). The green calli were generated solely in the presence of the auxin analog 2,4-D, bypassing the requirement for cytokinin that is normally needed for greening of yellow callus. When cytokinin was added to the green calli, shoot regeneration occurred with a higher frequency than from wild-type callus (Figures 1H–1J). Embryos, shoots, and roots of germinating T1 seedlings that constitutively expressed *ZmG2* or *ZmGLK1* were also greener than wild-type (Figures 1K–1P). Constitutive expression of *ZmG2* or *ZmGLK1* in rice is, therefore, sufficient to induce greening in tissues where chloroplasts don’t usually develop (callus, embryos, and roots) and to enhance greening in shoots.

### Maize *GLK* Gene Expression Significantly Increases the Volume of Functional Chloroplasts in Both Bundle Sheath and Mestome Sheath Cells of Rice

**Figure 2:**
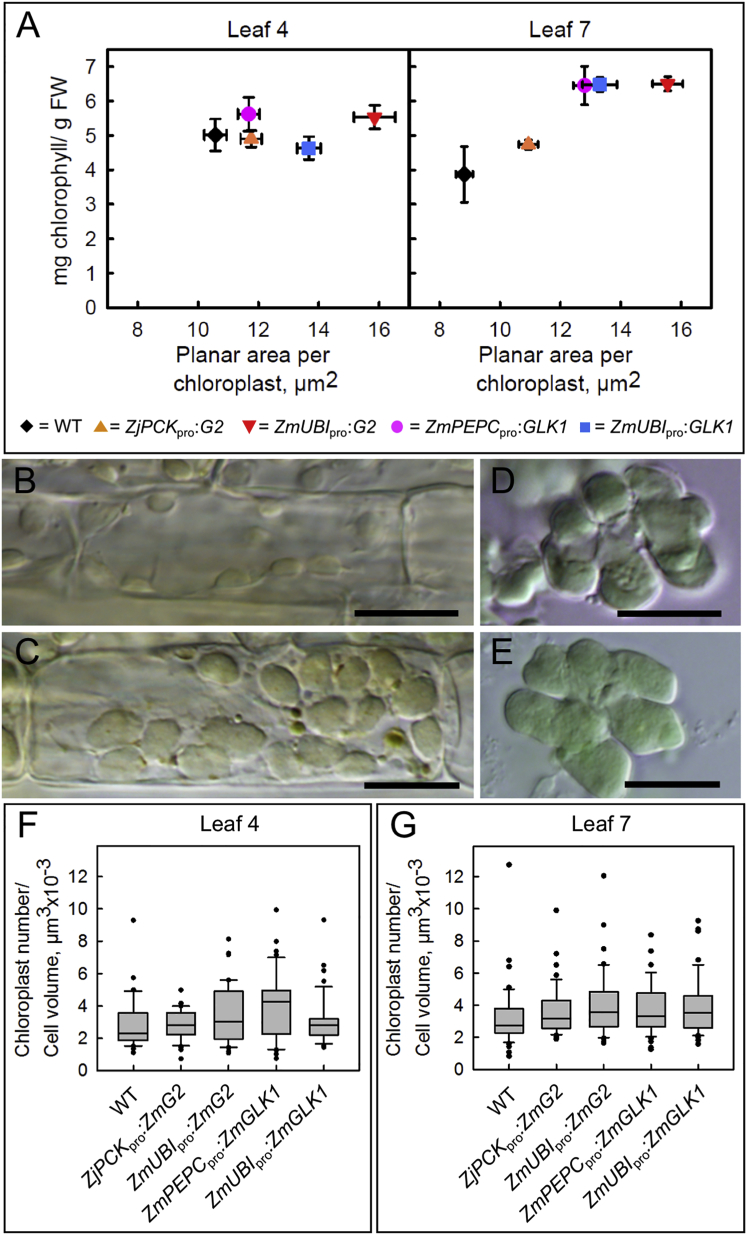
Chloroplast Size, but Not Number, Is Increased in Bundle Sheath Cells of *ZmG2* and *ZmGLK1* Overexpression Lines (A) Relationship between total leaf chlorophyll content and chloroplast planar area in bundle sheath cells of wild-type (WT) and T1 transgenic lines. Values are mean ± SEM (n = 3 individuals for chlorophyll content; n = 45 cells [15 cells/3 individuals] for chloroplast area). Chloroplast size was significantly increased in leaf 7 of all four transgenic lines relative to WT and in leaf 4 of lines constitutively expressing *ZmG2* or *ZmGLK1* (by a Games-Howell test of variance, p < 0.05). The phenotypic consequences of relatively weak transgene expression from the BS-preferential *ZjPCK*_pro_ were less dramatic than with the constitutive promoters. (B–E) Representative bundle sheath (B and C) and mesophyll (D and E) cells from WT (B and D) and *ZmUBI*_pro_:*ZmG2* (C and E) lines. Scale bars, 10 μm. (F and G) Boxplots showing chloroplast numbers in bundle sheath cells of leaf 4 (F) and leaf 7 (G) (n = 45 cells [15 cells from each of 3 individuals representing at least two independent lines] in each case). Boxplot whiskers indicate the range of observations. See Table S1 for details of transgenic lines used, Figure S1 for transgene copy number analysis, Figure S2 for transgene transcript levels, and Table S2 for a complete set of measurements.

The enhanced greening phenotype in transgenic rice lines expressing maize *GLK* genes could reflect increases in chloroplast size and/or number. To distinguish these possibilities, quantitative measurements were taken from the fourth and seventh leaves of cv. Kitaake lines that either constitutively expressed *ZmG2* or *ZmGLK1* or mimicked the expression profile observed in maize (namely, *ZmG2* expression preferentially in bundle sheath cells and *ZmGLK1* expression preferentially in mesophyll cells). Individual leaf samples were first analyzed for total chlorophyll levels (Figure 2A) and for both transgene and endogenous *OsGLK1/2* transcript levels (Figure S2). Both chloroplast size and number were then measured in bundle sheath cells that were isolated from the same leaves (Figure 2; Table S2). Chloroplast size was significantly increased in bundle sheath cells relative to wild-type at both stages of development and when either *ZmG2* or *ZmGLK1* was constitutively expressed (Figures 2A–2E; Table S2). Intriguingly the size increase induced by ZmGLK1 was manifest regardless of whether gene expression was driven by the constitutive *ZmUBI*_pro_ or the mesophyll preferential *ZmPEPC*_pro_. This suggests either that the effect on bundle sheath chloroplast size was induced non-cell autonomously or that the *ZmPEPC*_pro_ drove enough gene expression in the bundle sheath cells to have a phenotypic effect. Given that GLK proteins are known to act cell autonomously in *Arabidopsis* [33], it is most likely that the *ZmPEPC*_pro_ drives gene expression in rice bundle sheath cells but that transcript levels are so low that promoter activity has not been detected in previous reporter gene assays [32]. Quantification of chloroplast numbers per bundle sheath cell and of bundle sheath cell volume revealed no statistical difference between wild-type and any of the transgenic lines (Figures 2F and 2G; Table S2). Enhanced greening in transgenic lines is thus associated with an increase in chloroplast size that is unaccompanied by a decrease in chloroplast number, resulting in a larger chloroplast volume per bundle sheath cell relative to wild-type.

**Figure 3:**
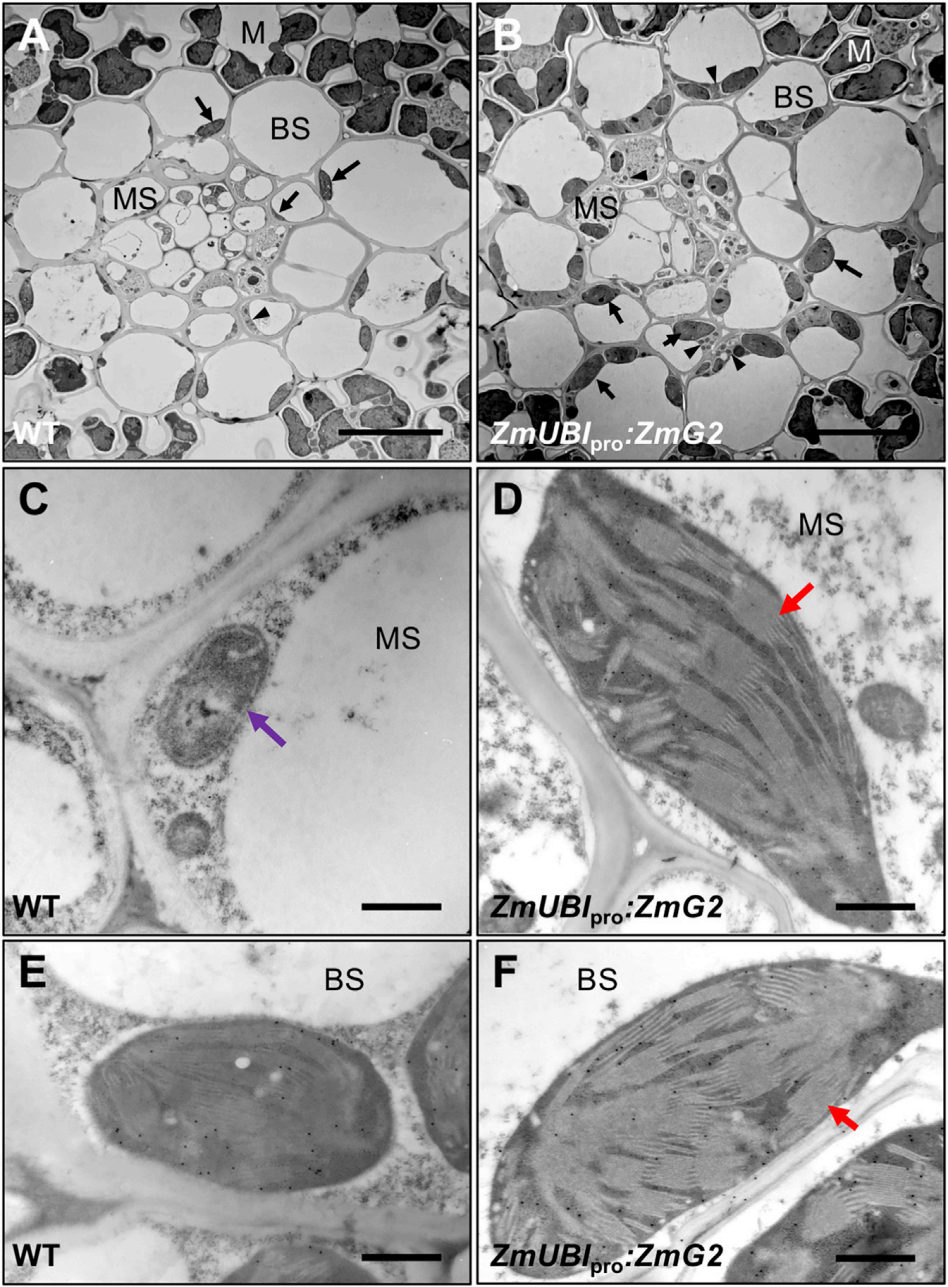
Chloroplasts in Both Bundle Sheath and Mestome Sheath Cells of Lines that Constitutively Express *ZmG2* Accumulate Photosynthetic Enzymes (A and B) Transmission electron micrographs of minor veins in leaf 7 of wild-type (WT) (A) and *ZmUbi*_pro_:*ZmG2* transgenic (B) plants grown at 300 μmol photons m^−2^ s^−1^. The position of bundle sheath (BS), mestome sheath (MS), and mesophyll (M) cells is indicated, with arrows pointing to representative chloroplasts and arrowheads pointing to mitochondria in each cell type. (C–F) Transmission electron micrographs of WT (C and E) and *ZmUbi*_pro_:*ZmG2* transgenic (D and F) lines showing plastid ultrastructure and immuno-gold labeling with RuBisCo antibody in MS (C and D) and BS (E and F) cells. Purple arrow, proplastid; red arrows, organized thylakoid stacks. Scale bars, 2 μm (A and B) and 500 nm (C–F). See Table 1 for quantification of immuno-gold labeling and Figure S3 for immuno-labeling with additional enzymes. See also Figures S1 and S2 and Tables S1 and S3.

Transmission electron microscopy (TEM) of leaf sections from wild-type (Figure 3A) and *ZmUBI*_pro_*ZmG2* transgenic **(**Figure 3B) lines confirmed that bundle sheath chloroplast volume is increased when *ZmG2* is expressed and revealed a similar distinction in mestome sheath cells (Figures 3A and 3B; Table S2). In wild-type leaves, only small undifferentiated plastids (proplastids) are found in mestome sheath cells (Figure 3C), but highly organized thylakoid systems were apparent in mestome sheath chloroplasts of *ZmUBI*_pro_*:ZmG2* leaves (Figure 3D), with the area of chloroplast coverage per cell being equivalent to that seen in the bundle sheath cells (Table S3). Mesophyll cell chloroplast size was indistinguishable between wild-type and transgenic lines (Table S3). To determine whether enhanced chloroplast development in vascular sheath cells was accompanied by the accumulation of photosynthetic enzymes, antibodies against RuBisCo, RuBisCo activase, and fructose-1,6-bis-phosphatase were reacted with tissue sections. Notably, all three enzymes could be detected in both bundle sheath and mestome sheath chloroplasts of *ZmUbi*_pro_*:ZmG2* transgenic plants (Figures 3C–3F; Figure S3), with levels in mestome sheath cells being significantly higher than in wild-type (Table 1). Constitutive expression of *ZmG2* in rice is thus sufficient to induce the development of both bundle sheath and mestome sheath chloroplasts with photosynthetic capacity.

**Table 1:** Quantification of Photosynthetic Enzymes in Vascular Sheath Cells

Cell Type	Transgene	Density of Gold Labeling/Planar Chloroplast Area (μm^−2^)		Density of Gold Labeling/Planar Cell Area (μm^−2^)	
		RuBisCo	RuBisCo Activase	RuBisCo	RuBisCo Activase
BS	WT	14.3 ± 0.6	52.4 ± 2.2	0.7 ± 0.1	2.7 ± 0.5
BS	*ZmUBI*_pro_:*ZmG2*	13.8 ± 0.5	59.0 ± 2.3	1.9 ± 0.6	7.7 ± 2.4
MS	WT	−	−	0.0	0.0
MS	*ZmUBI*_pro_:*ZmG2*	14.0 ± 0.7	64.2 ± 3.6	12.8 ± 3.7	51.7 ± 10.2

Mean ± SEM for 15 bundle sheath (BS) cells from leaf 7 of each of three transgenic *ZmUBI*_pro_:*ZmG2* individuals (representing 3 independent transgenic lines) and two wild-type (WT) individuals is given. The density of RuBisCo and RuBisCo activase is statistically similar per chloroplast planar area by a two-tailed t test (p ≥ 0.05). However, the significantly higher percentage of BS cell area covered by chloroplasts (Table S4; p < 0.05) translates into a 2- to 3-fold increase in the density of RuBisCo and RuBisCo activase per BS cell in *ZmUBI*_pro_:*ZmG2* lines relative to WT. More substantial increases are observed in mestome sheath (MS) cells. See also Figures S2 and S3 and Table S3.

### GLK-Induced Chloroplast Development in Rice Vascular Sheath Cells Is Accompanied by Enhanced Biogenesis of Mitochondria and Plasmodesmata

**Figure 4:**
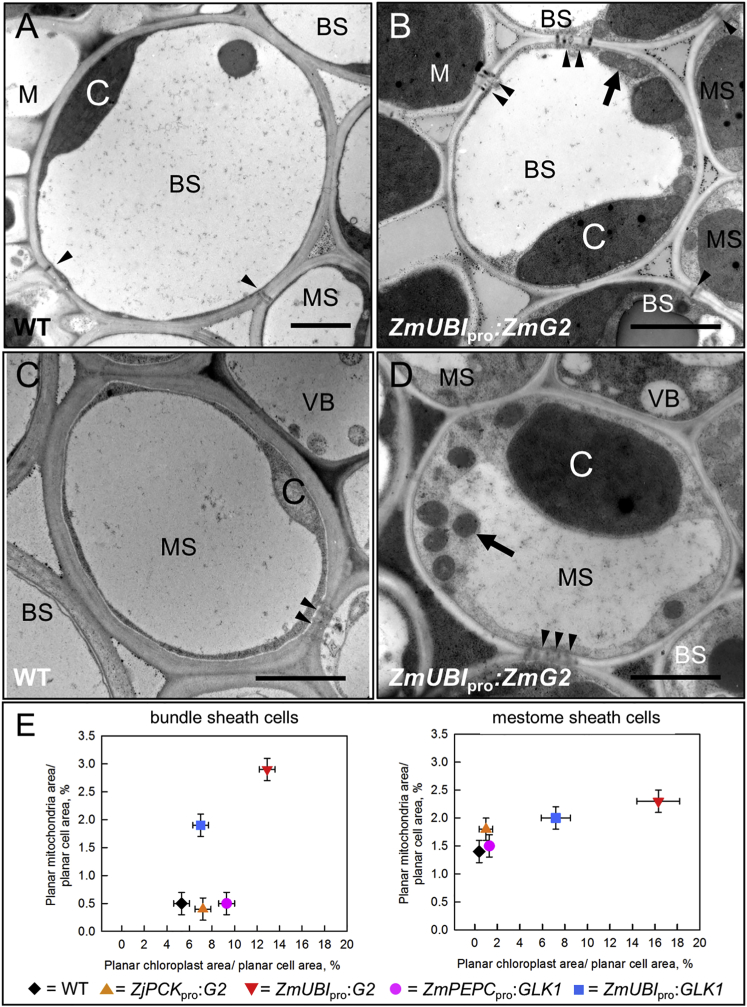
Chloroplast Development in Vascular Sheath Cells Is Accompanied by Increased Mitochondrial Volume and Plasmodesmatal Frequency (A–D) Transmission electron micrographs of bundle sheath (A and B) and mestome sheath (C and D) cells around a minor vein of leaf 7 in wild-type (WT) (A and C) and *ZmUBI*_pro_:*ZmG2* transgenic (B and D) lines. Arrows, mitochondria; arrowheads, plasmodesmata; BS, bundle sheath; M, mesophyll; MS, mestome sheath; VB, vascular bundle; C, chloroplast. Scale bars, 500 nm. (E) Quantification of relationship between chloroplast and mitochondrial planar area in vascular sheath cells; values are mean ± SEM (n = 45 [15 cells/3 individuals]). Percentage of cell area occupied by mitochondria is significantly increased relative to WT in both BS and MS cells of lines constitutively expressing *ZmG2* and in BS cells of lines constitutively expressing *ZmGLK1* (as judged by a Games-Howell test of variance, p < 0.05). See Table S3 for a complete set of organelle measurements and Table S4 for quantification of plasmodesmatal frequencies. See also Figures S1 and S2 and Table S1.

Qualitative analysis of TEM sections indicated that mitochondrial size and the frequency of plasmodesmata between cells were both increased in bundle sheath and mestome sheath cells of transgenic lines that constitutively expressed *ZmG2* (Figures 4A–4D). Quantification of mitochondrial size confirmed this suggestion and also demonstrated that mitochondrial number did not differ between wild-type and transgenic lines (Figure 4E; Tables S3 and S4). As with chloroplasts, the significant increase in mitochondrial volume represented enhanced functional capacity, as evidenced by accumulation of the photorespiratory glycine decarboxylase (Figure S3). Plasmodesmatal junctions were also significantly increased, both between bundle sheath and mesophyll and between bundle sheath and mestome sheath cells (Table S4). Notably, C_4_ grasses also exhibit higher plasmodesmatal frequencies between bundle sheath and mesophyll cells than C_3_ relatives [34]. Collectively, these data suggest that *ZmG2* activity can induce both photosynthesis and photorespiration in rice vascular sheath cells and that functionalization leads to greater plasmodesmatal connectivity between cell types.

### The Anatomical Traits Induced by Constitutive *ZmG2* or *ZmGLK1* Expression in Rice Mimic Those Seen in Proto-Kranz Species

**Figure 5:**
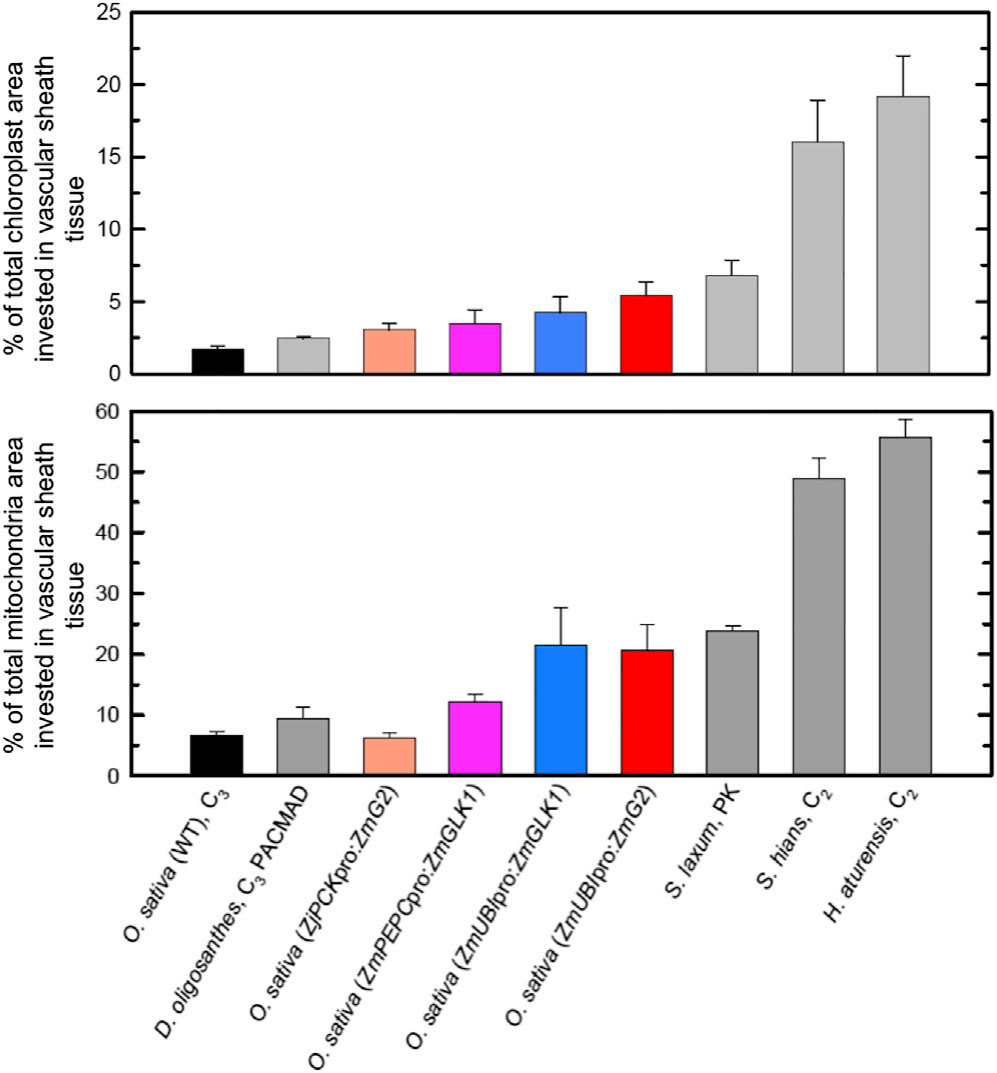
Percentage of Planar Organelle Area Invested in Vascular Sheath Cells Is Equivalent to that in Proto-Kranz Grass Species Percentage of total planar chloroplast (top) and mitochondrial (bottom) area in vascular sheath cells of wild-type (WT) and transgenic rice lines, plus PACMAD grasses *Dicanthelium oligosanthes* (C_3_), *Steinchisma laxum* (proto-Kranz [PK]), *S. hians* (C_2_), and *Homolepis aturensis* (C_2_). Values are mean ± SEM (n = 3). The trend toward proto-Kranz correlates with levels of transgene expression. Both *ZmUBI*_pro_*:ZmG2* and *S. laxum* (PK) have significantly greater investment of chloroplasts and mitochondria in vascular sheath tissue than WT by a one-tailed t test (p < 0.05). No significant differences were observed between *ZmUBI*_pro_*:ZmG2* and *S. laxum* (PK) by one-tailed or paired t tests (p > 0.2). See Figures S1 and S2 and Table S1 for transgene details and transcript levels in lines examined, Table S5 for a complete set of measurements, and Figure S4 for equivalent graphs of C_3_, PK, and C_2_*Flaveria spp*.

To assess the extent to which the vascular sheath cell phenotype of transgenic rice lines represents a transition along the C_3_-to-C_4_ trajectory, organelle composition was quantified in C_3_, proto-Kranz, and C_2_ species of both grass and eudicot clades. In proto-Kranz species, 4%–9% of the chloroplast area in the leaf and 18%–32% of the mitochondrial area is invested in vascular sheath cells (bundle sheath plus mestome sheath), which in grasses represents a 2.7-fold (chloroplasts) and 2.5-fold (mitochondria) enhancement relative to C_3_ species in the same clade (Figure 5; Figure S4; Table S5). Similarly, *ZmUBI*_pro_:*ZmG2* and *ZmUBI*_pro_:*ZmGLK1* transgenic lines have 4%–6% of leaf chloroplast area and 21% of leaf mitochondria area invested in vascular sheath cells, a 2.5- to 3-fold increase relative to wild-type (Figure 5; Table S5). As seen in other examples where C_3_ and proto-Kranz species have similar vascular sheath cell sizes (e.g., [14, 15]), increased organelle area in the transgenic rice lines was not associated with altered cell size. Collectively, these results demonstrate that constitutive expression of either *ZmG2* or *ZmGLK1* is sufficient to induce proto-Kranz anatomy in rice, recapitulating one of the earliest steps in C_4_ evolution.

### Proto-Kranz Anatomy in Rice Bears No Fitness Cost

**Figure 6:**
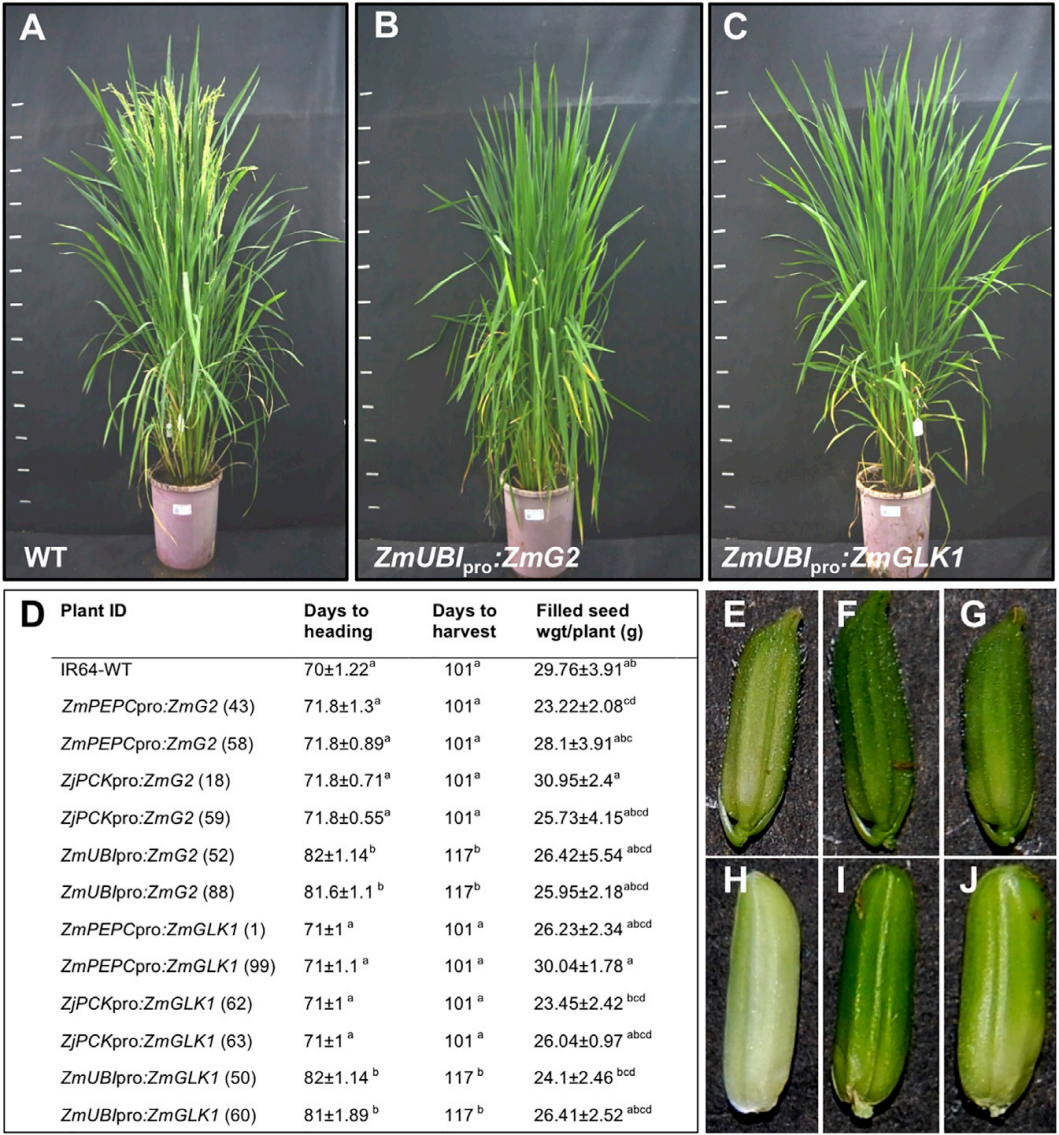
Constitutive Expression of *ZmG2* or *ZmGLK1* Delays Flowering but Has No Impact on Yield (A–C) Representative whole-plant phenotypes of wild-type (WT) (A), *ZmUBI*_pro_:*ZmG2* (B), and *ZmUBI*_pro_:*ZmGLK1* (C) IR64 lines photographed 75 days after sowing. (D) Heading (flowering) time and days to harvest (days after sowing in each case), plus filled seed weight (line number indicated in parentheses). Values are mean ± SEM of five individual plants. Means with the same letter are not significantly different, as assessed by ANOVA (p < 0.05). (E–J) Representative spikelet (E–G) and dehulled immature grain (H–J) phenotype of WT (E and H), *ZmUBI*_pro_:*ZmG2* (F and I), and *ZmUBI*_pro_:*ZmGLK1* (G and J) IR64 lines. See Figure S1 and Table S1 for transgene details and copy number analysis, Figure S5 for transgene transcript levels, and Figure S6 for photosynthetic measurements.

To determine whether the proto-Kranz anatomy that is induced by expression of *ZmG2* or *ZmGLK1* genes in rice has an impact at the whole-plant level, assays were conducted to measure photosynthetic efficiency and yield. These experiments were conducted in near-field conditions in the Philippines using cv. IR64 wild-type plus transgenic lines, in which expression from both constitutive and cell type preferential promoters had been confirmed (Figure S5). In all cases the transgenic lines resembled segregating wild-type plants in terms of gross morphology (e.g., plant height and tiller number) (Figures 6A–6C), and rates of leaf photosynthesis in response to both increased intercellular CO_2_ concentration (A/Ci curve) and increased photosynthetically active radiation (light response curve) were not significantly different from wild-type (Figures S6A and S6B). Similarly, the photosynthetic efficiency of photosystem II (Fv/Fm) (Figure S6C) and the non-photochemical quenching (NPQ) response (Figure S6D) were both similar to wild-type.

While the failure to detect altered rates of photosynthesis was initially surprising, the proportion of vascular sheath to mesophyll tissue is low in rice leaves (Table S5), and it is further likely that CO_2_ fixed in vascular sheath cells is sourced from respiration rather than from diffusion through mesophyll cells and intercellular air spaces [35]. Regardless, prolonged photosynthetic activity was evidenced by a significant delay in both heading date and time to harvesting physiologically matured seeds (Figure 6D) and by enhanced greening of immature grains (Figures 6E–6J). Whereas similar delayed senescence phenotypes can lead to reduced yield [36], no consistent differences in the yield of viable seed were observed between wild-type and transgenic lines (Figure 6D). As such, there is no fitness cost of proto-Kranz anatomy in rice.

## Discussion

The evolutionary trajectory from C_3_ to C_4_ most likely occurred in a stepwise fashion, with the order of events differing between lineages [8, 16, 17, 37, 38]. Combined evidence from qualitative, quantitative, and modeling approaches suggests, however, that the unifying early step in all trajectories was the transition from C_3_ to proto-Kranz [9, 12, 14, 15]. To recapitulate this step, organellar volume and photosynthetic/photorespiratory enzyme content in sheath cells surrounding the leaf veins must be increased. Constitutive expression of maize *GLK* genes in rice caused ectopic chloroplast development in cells around the vascular bundle, with photosynthetic enzymes accumulating to elevated levels in both bundle sheath and mestome sheath cells (Figures 1, 2, and 3; Table 1; Figure S3; Tables S2 and S3). Activation of chloroplasts in these cell types was accompanied by an increase in mitochondrial size, a corresponding increase in levels of the photorespiratory enzyme glycine decarboxylase, and a higher frequency of plasmodesmatal connections with neighboring cells (Figure 4; Tables S3 and S4). These subcellular modifications did not alter photosynthetic parameters in optimal growth conditions, and they did not impact on yield (Figure 6; Figure S6). In combination, these results demonstrate that constitutive expression of maize *GLK* genes in rice is sufficient to induce the transition from C_3_ leaf anatomy to proto-Kranz (Figure 5) and that the transition bears no fitness cost.

GLK proteins are transcription factors that directly activate a large number of downstream target genes encoding chloroplast-localized or photosynthesis-related proteins [25]. The modified development of mitochondria and plasmodesmata in bundle sheath and mestome cells of *ZmGLK* overexpression lines, therefore, most likely occurred as an indirect consequence of activated chloroplast development. Several communication routes between chloroplasts and mitochondria have been proposed, with the best characterized being signaling via redox state (for example, [39, 40, 41]). A direct link between chloroplasts, mitochondria, and plasmodesmata has also been recognized with loss-of-function mutants in RNA helicases that are localized in mitochondria and chloroplasts having increased numbers of plasmodesmata [42, 43, 44, 45]. These observations suggest the presence of intracellular signaling mechanisms that coordinate the biogenesis of chloroplasts, mitochondria, and plasmodesmata. The level of *GLK* gene activity is similarly regulated by intracellular signaling from the plastid, both at the transcriptional [25] and post-transcriptional levels [27, 28, 29].

In the lines generated here, developmental suppression of transgene expression by post-transcriptional regulation is evident (Figures S2 and S5); however, organelle enhancement in vascular sheath cells was sustained throughout. The lack of enhanced chloroplast development in mesophyll cells (Table S3), despite constitutive gene expression, suggests that endogenous feedback mechanisms moderated *GLK* transgene function in cells that were already photosynthetically active. Such feedback regulation would also explain why there is no direct correlation between transgene transcript levels (Figure S2) and the degree of phenotypic alteration in vascular sheath cells (Tables S2–S5). It is notable, however, that when constitutively expressed, ZmG2 (which is expressed preferentially in bundle sheath cells of maize) consistently induced more substantial changes in vascular sheath cells than ZmGLK1 (which is expressed preferentially in mesophyll cells of maize) (Figures 2, 3, 4, and 5; Tables S2–S5). This observation endorses the suggestion that the duplication of *GLK* genes in C_4_ lineages facilitated neo-functionalization for cell-type-specific roles [19].

The complexity of the anatomical and biochemical changes needed for the C_3_-to-C_4_ transition appears seemingly incongruent with the multiple independent origins of the pathway [1]. However, the results presented here suggest that one of the earliest steps in C_4_ evolution, the transition from C_3_ to proto-Kranz, could have resulted from modified activity of a single gene. During the course of land plant evolution, the probability of a C_3_-to-proto-Kranz transition presumably depended on the number of genes able to induce that change. Very few transcriptional regulators of chloroplast development have been discovered, but functional analyses with the cytokinin GATA transcription factor CYTOKININ-REPSONSIVE GATA TRANSCRIPTION FACTOR 1 (CGA1) [36, 46] and *GROWTH-REGULATING FACTOR 5* (*GRF5*) [47] suggest that both genes can activate chloroplast development in non-photosynthetic tissues. Roles upstream (GRF5) [47] and downstream (CGA1) [48] of GLK position both genes as potential regulators of at least some aspects of the C_3_-to-proto-Kranz transition. Whether or not this potential is validated, with the discovery that modifications to the activity of a single gene can kick-start the C_3_-to-C_4_ transition, one of the most remarkable examples of convergent evolution becomes slightly less mysterious.

## STAR★Methods

### Key Resources Table

**Table:** 

REAGENT or RESOURCE	SOURCE	IDENTIFIER
**Antibodies**		

Rabbit polyclonal anti-GLDP	Martha Ludwig	[12]
Rabbit polyclonal anti-RuBisCo 557 kDa hexadecamer	Agrisera, Martha Ludwig	AS07 218; RRID: AB_1031802
Rabbit polyclonal anti-FBPase	Christine Raines	[49]
Rabbit monospecific anti-RuBisCo activase	Elizabete Carmo-Silva	[50]
18nm Colloidal Gold-AffiniPure Goat Anti-Rabbit IgG (H+L) (min X Hu,Ms,Rat Sr Prot) (EM Grade)	Cedarlane	Cat# 111-215-144

**Bacterial and Virus Strains**		

*E. coli* strain DH5α	Widely distributed	N/A
*A. tumefaciens* strain EHA105	Widely distributed	N/A
*A. tumefaciens* strain LBA4404	Widely distributed	N/A

**Chemicals, Peptides, and Recombinant Proteins**		

Glutaraldehyde 25%	Electron Microscopy Sciences (EMS)	Cat# 16210
Paraformaldehyde (20%)	EMS	Cat# 15713
Sodium cacodylate	EMS	Cat# 12300
Osmium tetroxide 4%	EMS	Cat# 19150
Low viscosity embedding kit (Dr. Spurr)	EMS	Cat# 14300
LRW (medium grade)	EMS	Cat# 14381
Pectinase from *Aspergillus niger*	Sigma-Aldrich	Cat# 17389

**Critical Commercial Assays**		

Gateway BP Clonase II enzyme mix	Invitrogen	Cat#11789020
Gateway LR Clonase II enzyme mix	Invitrogen	*Cat#11791100*
GoTaq Green Master Mix	Promega	M712
GoTaq G2 Green Master Mix	Promega	M7822
TURBO DNA-free Kit	Ambion	Cat#AM1907
SuperScript III Reverse Transcriptase	Invitrogen	Cat#18080093
TERRA PCR Direct polymerase mix	Clontech	Cat. No. 639270
LightCycler 480 SYBR Green I Master (Real time PCR assays)	Roche	#04707516001
SYBR Green PCR Master Mix	Applied Biosystems	Cat#4309155
PCR DIG Probe Synthesis Kit	Roche	Cat. No. 1 636 090
Random Primers DNA Labeling System	Invitrogen	Cat#18187013

**Experimental Models: Organisms/Strains**		

*Oryza sativa spp. indica* cultivar IR64	IRRI	N/A
*Oryza sativa spp. japonica* cultivar Kitaake	IRRI	N/A
*Oryza sativa* IR64*: ZjPCK::ZmG2*	This paper	IR64-IRS-784
*Oryza sativa* IR64*: ZmPEPC::ZmG2*	This paper	IR64-IRS-782
*Oryza sativa* IR64*: ZmUbi::ZmG2*	This paper	IR64-IRS-786
*Oryza sativa* IR64*: ZjPCK:: ZmGLK1*	This paper	IR64-IRS-785
*Oryza sativa* IR64*: ZmPEPC:: ZmGLK1*	This paper	IR64-IRS-783
*Oryza sativa* IR64*: ZmUbi:: ZmGLK1*	This paper	IR64-IRS-787
*Oryza sativa* Kitaake *ZjPCKpro:ZmG2*	This paper	57C1, 57C2, 57C3
*Oryza sativa* Kitaake *ZmUBIpro:ZmG2*	This paper	57E1, 57E2, 57E3, 57E4
*Oryza sativa* Kitaake *ZmPEPC:ZmGLK1*	This paper	58A1, 58A2, 58A3, 58A4
*Oryza sativa* Kitaake *ZmUBIpro:ZmGLK1*	This paper	58E1, 58E2, 58E3, 58E4, 58E5

**Oligonucleotides**		

See Table S6	N/A	N/A

**Recombinant DNA**		

cDNA of *ZmG2*	[20]	GenBank: AF318579
cDNA of *ZmGLK1*	[20]	GenBank: AF318580
Gateway donor vector	Thermo Fisher Scientific	pDONR207
Binary destination vectors	Julian Hibberd	pSC110, pSC210, and pSC310
Plasmid pSC11057A, pSC11058A, pSC21057C, pSC21058C, pSC31057E, pSC31058E	This paper	N/A

**Software and Algorithms**		

SigmaPlot 12.5	Systat Software	http://www.sigmaplot.co.uk/products/sigmaplot/produpdates/prod-updates18.php
SPSS 20	IBM SPSS Statistics	https://www.ibm.com/support/knowledgecenter/en/SSLVMB_20.0.0/com.ibm.spss.statistics_20.kc.doc/pv_welcome.html
ImageJ	NIH	https://imagej.nih.gov/ij/

**Other**		

Soil: John Innes Compost No. 2	Widely available	N/A
Miracle-Gro All Purpose Plant Food	https://www.miraclegro.com/	N/A

### Contact for Reagent and Resource Sharing

Further information and requests for resources and reagents should be directed to and will be fulfilled by the Lead Contact, Jane Langdale (jane.langdale@plants.ox.ac.uk). Please note that the transfer of transgenic rice lines will be governed by an MTA, will be dependent on appropriate import permits being acquired by the receiver, and may be constrained by the size of available seed stocks.

### Experimental Model and Subject Details

#### Plants

*Oryza sativa spp. indica* cultivar IR64 lines were grown in a transgenic greenhouse at the International Rice Research Institute (IRRI), Los Baños, Philippines (located at 14°9′53.58″S 121°15′32.19″E). The average day/night temperatures in the plant growth facility were 35 ± 3°C and 28 ± 3°C, respectively. Average light intensity and photoperiod were 824.75 ± 201.86 μmol photons m^-2^ s^-1^ and 13 hr day/11 hr night, respectively.

*Oryza sativa spp. japonica* cultivar Kitaake lines were grown in soil in a transgenic greenhouse in either Oxford, UK or Canberra, Australia. Day/night temperature was maintained at 30°C/22°C ± 3°C with a diurnal light regime of 16 hr light (supplemented to ∼300 μmol photons m^-2^ s^-1^) and 8 hr dark.

All plants were self-pollinated by bagging inflorescences after flowering.

#### Microbes

*Agrobacterium tumefaciens* strains LBA4404 and EHA105 were cultured at 28°C in LB plus 100 mg L^-1^ rifampicin and supplemented with 50 mg L^-1^ kanamycin if harboring a plasmid for rice transformation.

### Method Details

#### Gene cloning and construct design

Full-length cDNAs of *ZmG2* (GenBank: AF318579) and *ZmGLK1* (GenBank: AF318580) were amplified by PCR from cDNA clones isolated previously [20] using Gateway compatible primers ZmG2-cloningF, ZmG2-cloningR, ZmGLK1-cloningF and ZmGLK1-cloningR (Table S6). The coding sequences were subcloned into the Gateway donor vector pDONR207 via a BP reaction, and the resulting entry clones were sequenced. Coding sequences were then cloned downstream of the *ZmPEPC*, *ZjPCK*, and *ZmUBI* promoters in the binary destination vectors pSC110, pSC210, and pSC310, respectively. The maize *UBIQUITIN* promoter (*ZmUBI*_pro_) was used to drive constitutive expression [30], the *Zoysia japonica PHOSPHOENOLPYRUVATE CARBOXYKINASE* promoter (*ZjPCK*_pro_) for preferential expression in bundle sheath cells [31] and the maize *PHOSPHOENOLPYRUVATE CARBOXYLASE* promoter (*ZmPEPC*_pro_) for preferential expression in mesophyll cells [32]. Six constructs were produced via a LR reaction (Figure S1A).

#### Rice transformation

*Oryza sativa spp. indica* cultivar IR64 and *spp. japonica* cultivar Kitaake were both used for transformation. For IR64, immature embryos were isolated from freshly harvested spikelets 8-12 days after anthesis. Embryos were inoculated with *Agrobacterium tumefaciens* strain LBA4404 carrying the plasmid DNA of interest and then cultured and regenerated according to the protocol of Hiei and Komari [51]. Briefly, after one week of co-cultivation at 25°C in the dark followed by growth on non-selective medium for 5 days, emerging resistant calli were selected on 30 mg L^-1^ hygromycin B. Regeneration was subsequently carried out on 50 mg L^-1^ hygromycin B for two weeks. Hygromycin-resistant regenerated T0 plantlets were transferred to Yoshida hydroponics solution [52] for 2 weeks prior to transplanting into soil in 7 L pots. For Kitaake, calli induced from mature seeds were transformed with *A. tumefaciens* strain EHA105 carrying the construct of interest. Callus induction, transformant selection and seedling regeneration were performed at 32°C under continuous light according to a protocol modified from [53] that can be downloaded at https://langdalelab.files.wordpress.com/2015/07/kitaake_transformation_2015.pdf. Hygromycin-resistant T0 seedlings that screened positive for the transgene by PCR were transplanted into soil (John Innes Compost No.2) in 0.73 L pots.

#### PCR screening

Regenerated T0 plants were subjected to genomic PCR using primers specific to the gene of interest or the cloning vector (Table S6: pVec8F (*UBI*_pro_), pVec8R (nosT), pSC110F(*PEPC*_pro_), pSC210F(*PCK*_pro_), HPTFpr3, HPTRpr2, pSC1/2/310-R, ZmG2-F, ZmG2-R, ZmGLK1-F, ZmGLK1-R). For IR64 T0 lines, small leaf sections were harvested two weeks after regeneration and used directly as templates for PCR screening. For T1 seedlings, leaf samples were harvested 10 days after germination. PCR amplification was performed in a total reaction volume of 10 μL using TERRA PCR Direct polymerase mix (Clontech). Plasmid DNA was used as a positive control and non-transgenic rice DNA and water were separately used as negative controls. PCR conditions were as follows: initial-denaturation for 2 min at 98°C; 30 cycles of amplification consisting of denaturation for 10 s at 98°C, annealing for 15 s at 60°C and extension for 45 s at 68°C; followed by a final extension step for 7 min at 72°C. For Kitaake lines, genomic DNA was isolated using a modified cetyl trimethylammonium bromide (CTAB) method [54] (see below), and was tested in 10 μL PCR reactions containing 5 μL 2xGoTaq mix (Promega) and 2.5 μL 4M betaine. PCR conditions were: 95°C for 5 min; 28 cycles of 95°C for 30 s, 55°C for 40 s, 72°C for 2.5 min; and 72°C for 5 min.

#### DNA gel blot analysis

Genomic DNA was extracted from leaves of mature plants using potassium acetate (IR64 lines) [55] or CTAB (Kitaake lines) [54] methods. With the potassium acetate method, 1-2 g of rice leaves were ground to a fine powder in liquid nitrogen and suspended in 15 mL extraction buffer (100 mM Tris-HCl, 50 mM EDTA pH8.0, 500 mM NaCl, 0.07% β-mercaptoethanol). 1 mL 20% SDS was then added to the suspension, mixed well and incubated at 65°C for 30 min. After that time, 5 mL 5 M potassium acetate was added and mixed gently by shaking, before incubating at 4°C for 20 min. Samples were then centrifuged at 3500 rpm for 30 min, the supernatant was transferred to a fresh tube and nucleic acid was precipitated by adding 10 mL isopropanol followed by incubation at −20°C for 20 min. Nucleic acid was pelleted by centrifugation at 5000 rpm for 15 min and then re-suspended in 500 μL H_2_O. Samples were digested with 1 μL RNase (10 mg/mL) at 37°C for 10 min, extracted with 500 μL of chloroform: isoamyl alcohol (24:1) and centrifuged at 12000 rpm for 10 min. Supernatants were transferred to fresh tubes and DNA precipitated by adding 75 μL 3 M Sodium acetate and 500 μL isopropanol. After centrifugation at 12000 rpm for 5 min, pellets were washed twice with 500 μL 70% ethanol and air-dried. DNA pellets were dissolved in 100 μL H_2_O and stored at −20°C. With the CTAB method, leaves were first ground in liquid nitrogen, and suspended in 500 μL CTAB extraction solution containing 1.5% CTAB, 1.05 M NaCl, 75 mM Tris-HCl, 15 mM EDTA pH8.0. After incubation at 65°C for 20 min, an equal volume of chloroform:isoamylalcohol (24:1) was added, samples were mixed by vortexing and then centrifuged at 12000 rpm for 5 min. Supernatants were transferred to fresh tubes, precipitated by adding 2.5 volumes of 100% ethanol, and centrifuged at 12000 rpm for 5 min. The pellets were washed with 800 μL 70% ethanol, and air-dried. DNA samples were dissolved in 50 μL H_2_O and stored at −20°C.

For each plant, 6-8 μg of genomic DNA was digested with *Afl* II (*ZmG2* samples) or *Hin*d III (*ZmGLK1* samples) restriction endonuclease. Digested DNA was electrophoresed and then transferred onto Hybond N+ membrane (GE Healthcare, UK). For IR64 lines, blots were hybridized with digoxygenin (DIG)-labeled gene specific probes synthesized using primers indicated in Figure S1A and Table S6, and the PCR DIG Probe Synthesis Kit (Roche Diagnostics, Germany). Hybridization signals were detected using CDP-Star according to the manufacturer’s instructions (Roche Diagnostics). For the Kitaake lines, blots were hybridized as in [56], with ^32^P labeled fragments of the hygromycin resistance gene synthesized using the Random Primers DNA Labeling System (Invitrogen), and exposed to autoradiography film.

Two independent IR64 lines with one or two transgene copies were obtained for each of the six constructs (Figures S1B and S1C; Table S1) and at least two independent Kitaake lines, with transgene copy numbers ranging from one to four, were obtained for *ZjPCK*_pro_:*ZmG2, ZmUBI*_pro_:*ZmG2, ZmPEPC*_pro_:*ZmGLK1* and *ZmUBI*_pro_:*ZmGLK1* constructs (Figures S1D–S1G; Table S1).

#### Quantitative RT-PCR

4^th^ and 7^th^ leaves were harvested from transgenic plants and wild-type controls when leaves were at the youngest fully expanded stage. RNA was extracted using TRIZOL reagent (Invitrogen) or a QIAGEN RNAeasy kit, and RNA integrity was confirmed by gel electrophoresis. For IR64 lines, total RNA was treated with RQ1 RNase free DNase (Promega) prior to use as template to synthesize cDNA with a first strand cDNA synthesis kit (Roche Diagnostics). Quantitative real time PCR was carried out with LightCycler 480 SYBR Green I Master mix in a final reaction volume of 20 μL (Roche Diagnostics). Primers specific to the gene of interest and actin (Table S6) were used. For Kitaake lines, TURBO DNA-free Kit (Ambion) was used for DNase treatment, *SuperScript III* Reverse Transcriptase (Invitrogen) was used for first strand cDNA synthesis, and SYBR Green PCR Master Mix (Applied Biosystems) was used for quantitative RT-PCR on a StepOnePlus System (Applied Biosystems).

#### Chlorophyll quantification

Leaf tissues of equal fresh weight were ground to a fine powder in liquid nitrogen, prior to submergence in equal volumes of 80% acetone. Samples were incubated overnight in the dark at 4°C prior to centrifugation for 1 min at 15,000 x g to remove cell debris. The chlorophyll content of the supernatant was measured at 663 and 645 nm using a spectrophotometer and quartz cuvettes. Total chlorophyll extracted (μg mL^-1^) was calculated as follows: (8.02 × Absorbance@663 nm) + (20.29 × Absorbance@645 nm) [57], and adjusted per weight of fresh tissue sampled (g).

#### Isolation of single cells

2 mm sections of the recently fully expanded mid-region of leaves 4 and 7 were fixed (1 h) in 0.5% glutaraldehyde in 0.1 M sodium cacodylate buffer and incubated (3 h) in 0.2 M disodium EDTA, pH 9.0 in a water bath at 55°C. After incubation, samples were rinsed (20 min) in water and subsequently digestion buffer (20 min; 0.15 M sodium hydrogen phosphate, 0.04 M citric acid, pH 5.3). Leaf tissues were then incubated (1 h) at 45°C in 2% pectinase in digestion buffer and rinsed (30 min) in digestion buffer. Bundle sheath cells could be distinguished from mesophyll cells by an elongated shape (Figures 2B and 2D). Isolated bundle sheath cells were viewed with Nomarski optics and bundle sheath cell volume and chloroplast size were quantified with ImageJ [58] from images captured on a Zeiss Axioplan equipped with Olympus cellSens imaging software.

#### Light and transmission electron microscopy

Leaves 4 and 7 were prepared for light and transmission electron microscopy (TEM), quantification of cellular features, and immunodetection of photosynthetic enzymes and glycine decarboxylase as described previously [59]. 2 mm leaf sections from the middle of recent fully expanded leaves were fixed in 1% glutaraldehyde and 1% paraformaldehyde in 0.1 M sodium cacodylate buffer (pH 6.8) overnight at room temperature, and post-fixed (2 hr) in 1% osmium tetroxide. Fixed tissue was rinsed (2 × 30 min) in 0.1 M sodium cacodylate buffer. Samples were subsequently dehydrated in ethanol:H_2_O with 10% increment increases from 10% to 100% ethanol (1 hr each increment) and two (1 hr each) changes of 100% ethanol. Dehydrated tissue samples were infiltrated in Spurr’s resin using 10% increment increases of Spurr’s in 100% ethanol (3 hr each increment) from 10 to 100% Spurr’s. After two changes in 100% Spurr’s (12 hr each), tissue was polymerized at 60°C in a flat embedding mold. Fixation of leaf tissue for immunodetection of enzymes followed this same protocol except post-fixation with osmium tetroxide was omitted and specimens were infiltrated in London Resin White (LRW) using 1:3, 1:1, and 3:1 ratio of LRW to 100% ethanol (8 hr each increment), followed with 2 × 100% LRW (8 hr each). LRW infiltrated leaf samples were polymerized at 60°C in an oxygen-free environment for 12 hr at 60°C. Sections of 1.7 μm and 50-70 nm were collected for light and TEM, respectively. Images for light microscopy were captured on a Ziess Axioplan equipped with Olympus cellSens imaging software and a Phillips 201 transmission electron microscope equipped with an Advantage HR camery system (Advanced Microscopy Techniques) was used to capture transmission electron micrographs.

#### Immunohistochemistry

50-70 nm sections of LRW embedded tissue were rehydrated in 0.01 M phosphate saline buffer (PBS) pH. 7.4, blocked for 15 min with 0.4% or 0.5% bovine serum albumin (BSA) in PBS (for glycine decarboxylase and photosynthetic enzyme detection respectively), and then rinsed in PBS for 3 × 15 min before incubating for 3 hr in primary antibody at the following concentrations: 1:50 (anti-glycine decarboxylase), 1:100 (anti-RuBisCo and anti-RuBisCo activase), and 1:400 (anti-FBPase) in 0.1% BSA/PBS. Sections were then rinsed in PBS 3 × 15 min before incubation with secondary antibody (18nm Colloidal Gold-AffiniPure Goat Anti-Rabbit IgG) for 1 hr at a concentration of 1:20 (glycine decarboxylase) or 1:40 (photosynthetic enzymes) in 0.1% BSA/PBS. Samples were then rinsed in PBS for 3 × 15 min, followed by ultrapure water for 3 × 15 min before being stained with 4% uranyl acetate for 10 min, and then lead citrate for 5 min [60].

#### Gas exchange measurements

Fully expanded 8^th^ leaves were used to measure photosynthetic rates using a LI-6400XT porTable photosynthesis system (LICOR Biosciences). Three individuals were sampled per line, and measurements were repeated three times. Measurements were made in the morning at a constant airflow rate of 400 μmol s^-1^, leaf temperature of 30°C and a leaf-to-air vapor pressure deficit of between 1.0 and 1.5 kPa. Leaves were acclimated in the chamber for approximately 30 min before measurements were made on the mid-portion of the leaf blade. Net CO_2_ assimilation rate (*A*) in response to intercellular CO_2_ concentration (*Ci*) was measured at a light intensity of 2,000 μmol photons m^–2^ s^–1^ by increasing CO_2_ concentration in the cuvette from 20 to 2,000 μmol CO_2_ mol^–1^ air. Similarly, light response curves were produced by measuring net CO_2_ assimilation rate (*A*) in response to increasing photosynthetic photon flux density (PPFD) from 20 to 2,000 μmol photons m^-2^ s^–1^ at a CO_2_ concentration of 400 μmol CO_2_ mol^– 1^ air.

#### Chlorophyll fluorescence for Fv/Fm and NPQ measurements

For each individual, the three youngest fully expanded leaves of plants at maximum tillering stage were cut at the base and placed on a tray with the base of the leaves soaked in water to prevent drying and folding. Three individuals were sampled per line. The samples were dark adapted for 30 min prior to measurement. Fv/Fm and non photochemical quenching (NPQ) measurements were carried out using the PlantScreen Phenotyping System from Photon Systems Instruments (PSI), Czech Republic. Fluorescence images captured by the fluorometer were analyzed using the FluorCam7.0 software (PSI, Czech Republic).

### Quantification and Statistical Analysis

#### Experimental design, sampling and statistical methods

For all experiments, at least three individuals representing at least two independent transgenic lines were evaluated. Experiments to measure photosynthetic parameters and seed yield were carried out using wild-type and transgenic lines of *Oryza sativa spp. indica* cultivar IR64 (Figure 6; Figures S1, S5, and S6; Table S1). All other experiments were carried out with wild-type and transgenic lines of *Oryza sativa spp. japonica* cultivar Kitaake.

To assess the impact of developmental regulation: samples harvested from leaf 4 and leaf 7 of plants grown in the same environment were compared, measuring transgene transcript levels in the same leaves used for phenotypic analysis. For qRT-PCR (Figure S2) and TEM/immunolocalization (Figures 3, 4, and 5; Table 1; Figure S3; Tables S3–S5) experiments, samples were collected 3 hr post-illumination. To minimize interference from starch granules, the same leaf was sampled 20 hr later for single cell measurements (Figure 2; Table S2).

For quantification of heading date and seed weight, five individuals from each of two independent lines per construct were analyzed (Figure 6). Transgene transcript levels were quantified in leaf 4 and 7 of three individuals from each of those independent lines (Figure S5), and measurements of photosynthetic capacity were carried out in leaf 8 of three individuals from one of those lines for each construct (Figure S6).

qRT-PCR experiments were carried out using three experimental replicates per biological sample (Figures S2 and S5). The 2^-ΔCT^ method was used to quantify the relative abundance of transcripts using the CT value of *OsActin* as the internal control for normalization [61]. Primer pairs for *ZmGLK*s, *OsGLK*s and *OsActin* were tested in standard PCR reactions against maize and rice cDNA respectively to ensure specific targets were amplified. For each primer pair, a standard curve was generated to ensure amplification efficiency had a linear relationship with cDNA concentration, with all primer pairs giving a linear regression (*R*^2^) value of 0.99, except for the primer pair for *OsGLK2* that showed an *R*^2^ value of 0.93. The overall primer efficiency values were between 96.8%–108.1%.

Average of individual chloroplast area and chloroplast numbers in isolated cells were quantified for 15 bundle sheath cells per individual (Figure 2; Table S2). 15 was established as the sample size based on preliminary experiments which showed that using more than 15 failed to alter the mean and variance for each individual. Three individuals were used per line as a standard minimum requirement. Bundle sheath cell volume was approximated using cell volume = [(W/2)^2^]. π.(L), where W = planar cell width and L = planar cell length. Cell volume was then used to quantify the number of chloroplasts/cell volume.

Planar cell area covered by total chloroplasts and mitochondria was quantified using ImageJ, sampling 15 mesophyll, bundle sheath or mestome sheath cells from TEM images of leaf 7 for each of three individuals from *ZjPCK*_pro_:*ZmG2, ZmPEPC*_pro_:*ZmGLK1, ZmUBI*_pro_:*ZmG2*, and *ZmUBI*_pro_:*ZmGLK1* lines (Figures 2 and 4; Table S3). Similar leaf tissue and the same sample size was used for quantification of gold density on *ZmUBI*_pro_:*ZmG2* and wild-type sections immuno-labeled for RuBisCo and RuBisCo activase (Table 1; Figure S3). The same leaf materials were used to quantify the percentage of leaf cross section covered by mesophyll, bundle sheath, and mestome sheath cells. The percentage of organelles invested in each cell-type was then quantified as the proportion of organellar area in that cell-type, relative to the total photosynthetic organellar area (Figure 5; Figure S4; Table S5) (as in the three equations below):

1) Total planar area of photosynthetic tissue (TP) was quantified as:

$$\mathrm{TP}={\text{T}}_{\text{M}}+{\text{T}}_{\text{BS}}+{\text{T}}_{\text{MS}}$$

Where T = % cell-type area in cross section (T_M_ = % planar Mesophyll, T_BS_ = % planar Bundle Sheath, T_MS_ = % planar Mestome Sheath)

2) Total photosynthetic organellar area (PO) in planar cross section (%) was quantified as:

$$\text{PO}=\left[\left({\text{T}}_{\text{M}}/\text{TP}\right){\text{O}}_{\text{M}}\right]+\left[\left({\text{T}}_{\text{BS}}/\text{TP}\right){\text{O}}_{\text{BS}}\right]+\left[\left({\text{T}}_{\text{MS}}/\text{TP}\right){\text{O}}_{\text{MS}}\right]$$

Where O is the planar cell area covered by organelles (chloroplast or mitochondria)

3) The % of organelles invested in each cell-type was quantified as:

%oforganelleinvestment=CO/PO×100

Where CO is the % of TP occupied by the cell-type (M, BS, or MS) organelle area; e.g., CO_M_ = (T_M_/TP) O_M_

Similar quantifications were performed for six PACMAD grasses from the raw data in [12]. *Flaveria* organellar data were from [14, 15], and the values for cell type % in *Flaveria* species were extracted from M:BS ratios in Figure 4 of [62].

For all cellular measurements, statistical tests were performed using SPSS 20.0 and graphed using SigmaPlot 12.5 For continuous variables, a Shapiro-Wilk test of normality was used to determine whether the data were normally distributed. When the normality test failed, a General Linear Model (GLM) followed by a Games-Howell post hoc test was performed. An ANOVA and a Tukey’s test was used for normally distributed data. Presence or absence of plasmodesmata between bundle sheath-mesophyll and between bundle sheath-mestome sheath cells was recorded as absence = 0, presence = 1. Relative frequency of plasmodesmata was calculated by dividing the number of cells which had plasmodesmatal connections with the adjacent cell with the total number of observed cells for that cell-type. A GLM for binary data was performed where presence and absence of plasmodesmata between cell-types was the dependent variable, line was the fixed factor, and bundle sheath planar chloroplast and mitochondria area per planar cell area were covariates, using line as the main effect and a confidence interval of 95%.

Standard errors for heading time, days to harvest and filled seed weight, and ANOVA were calculated using the Statistical Tool for Agricultural Research (STAR) 2.01 software.

## Author Contributions

Experimental Concept and Design, P.W., R.K., S.K., W.P.Q., R.F., T.L.S., and J.A.L.; Generation of Transformation Constructs and Genotypic, qPCR, and Phenotypic Analyses of Kitaake Lines, P.W.; Generation of IR64 Lines and Genotypic and Phenotypic Analyses of IR64 Lines, S.K.; Organelle Counts and TEM Analysis, R.K.; qPCR of IR64 Lines, R.T., C.P.B., and S.K.; DNA Gel Blot Analysis of IR64 Lines, C.P.B.; Data Analysis and Interpretation, P.W., R.K., S.K., A.B., W.P.Q., R.F., T.L.S., and J.A.L.; Initial Manuscript Preparation, P.W., R.K., S.K., T.L.S., and J.A.L. All authors contributed to the final version of the manuscript.

## Supplemental Information

Document S1. Figures S1–S6 and Tables S1–S6

Document S2. Article plus Supplemental Information
