# Supplementary material for: Re-creation of a Key Step in the Evolutionary Switch from C3 to C4 Leaf Anatomy
Source: Curr Biol. 2017 Nov 6;27(21):3278–3287.e6. doi: 10.1016/j.cub.2017.09.040 (PMC5678070; doi:10.1016/j.cub.2017.09.040)
Supplement: Document S1. Figures S1–S6 and Tables S1–S6 [file mmc1.pdf]

Current Biology, Volume *27*

Supplemental Information

Re-creation of a Key Step in the Evolutionary Switch from C_3 to C_4 Leaf Anatomy

Peng Wang, Roxana Khoshravesh, Shanta Karki, Ronald Tapia, C. Paolo Balahadia, Anindya Bandyopadhyay, W. Paul Quick, Robert Furbank, Tammy L. Sage, and Jane A. Langdale

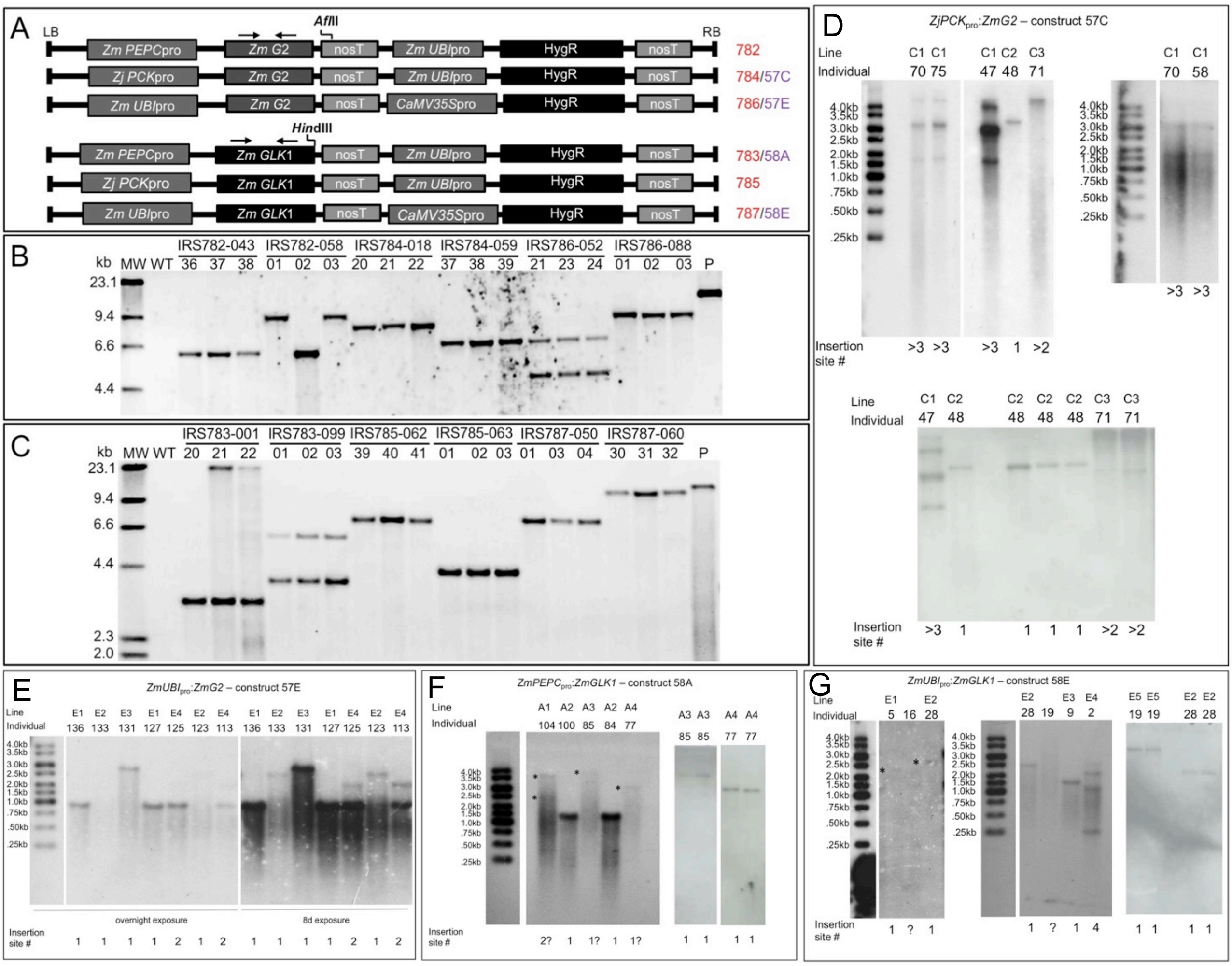

Figure S1. Transgene copy number in *ZmG2* and *ZmGLK1* lines. Related to Figures 1-6.

A) Constructs used for transformation. Arrows show primer positions used to amplify fragments for hybridization in (B) & (C). Blots in (D) - (F) were hybridized with the hygromycin resistance gene. Single *Afl* II and *Hind* III restriction sites within the constructs are indicated. LB = left border; RB = right border of T DNA. Numbers in red indicate construct reference for transformation into *O. sativa spp. indica* cultivar IR64, and in purple indicate construct reference for transformation into *O. sativa spp. japonica* cultivar Kitaake. **B, C)** Gel blot analysis of IR64 lines. *Afl* II digested DNA from wild-type (WT) plus 3 individuals of each of 2 independent *ZmPEPC*$_{pro}$:*ZmG2* (IRS782-043; -058), *ZjPCK*$_{pro}$:*ZmG2* (IRS784-018; -059) & *ZmUBI*$_{pro}$:*ZmG2* (IRS786-052; -088) T1 lines. All lines have individuals with a single transgene insertion site except for IRS786-052 which has two. In line IRS782-058, either two transgenes have segregated or there is a 3kb deletion in one of the three individuals (B). *Hind* III digested DNA from WT plus 3 individuals of 2 independent *ZmPEPC*$_{pro}$:*ZmGLK1* (IRS783-001; -099), *ZjPCK*$_{pro}$:*ZmGLK1* (IRS785-062; -063) & *ZmUBI*$_{pro}$:*ZmGLK1* (IRS787-050; -060) T1 lines. All lines have at least one individual with a single transgene insertion site except for IRS783-099 which has two. In line IRS783-001, segregation of two transgenes has resulted in one individual that is homozygous for one transgene (20), one that is homozygous for both transgenes (21) and one that is homozygous for one transgene and hemizygous for the second (22) (C). MW – molecular weight markers; P – plasmid. **D-G)** Cropped images of gel blot analysis of Kitaake lines. *Afl* II digested DNA from T1 plants (top) & T2 progeny (bottom) of lines transformed with *ZjPCK*$_{pro}$:*ZmG2* construct 57C. 6 individuals were derived from 3 independent transgenic events, one of which (C2) represents a single insertion site (D). *Afl* II digested DNA of T1 lines transformed with *ZmUBI*$_{pro}$:*ZmG2* construct 57E. 7 individuals display 4 independent transgene profiles, three of which (E1-E3)

represent a single insertion site (E). *Hind* III digested DNA of T1 plants (left) & T2 progeny (middle & right) of lines transformed with *ZmPEPC*$_{pro}$:*ZmGLK1* construct 58A. 5 individuals display 4 independent transgene profiles, three of which (A2, A3 & A4) represent a single insertion site (F). *Hind* III digested DNA of T1 plants (left & middle) and T2 progeny (right) lines transformed with *ZmUBI*$_{pro}$:*ZmGLK1* construct 58E. 6 individuals display at least 5 different transgene profiles, at least three of which (E2, E3 & E5) represent a single insertion site (G). Asterisks are positioned to the left of faint bands in (F) & (G).

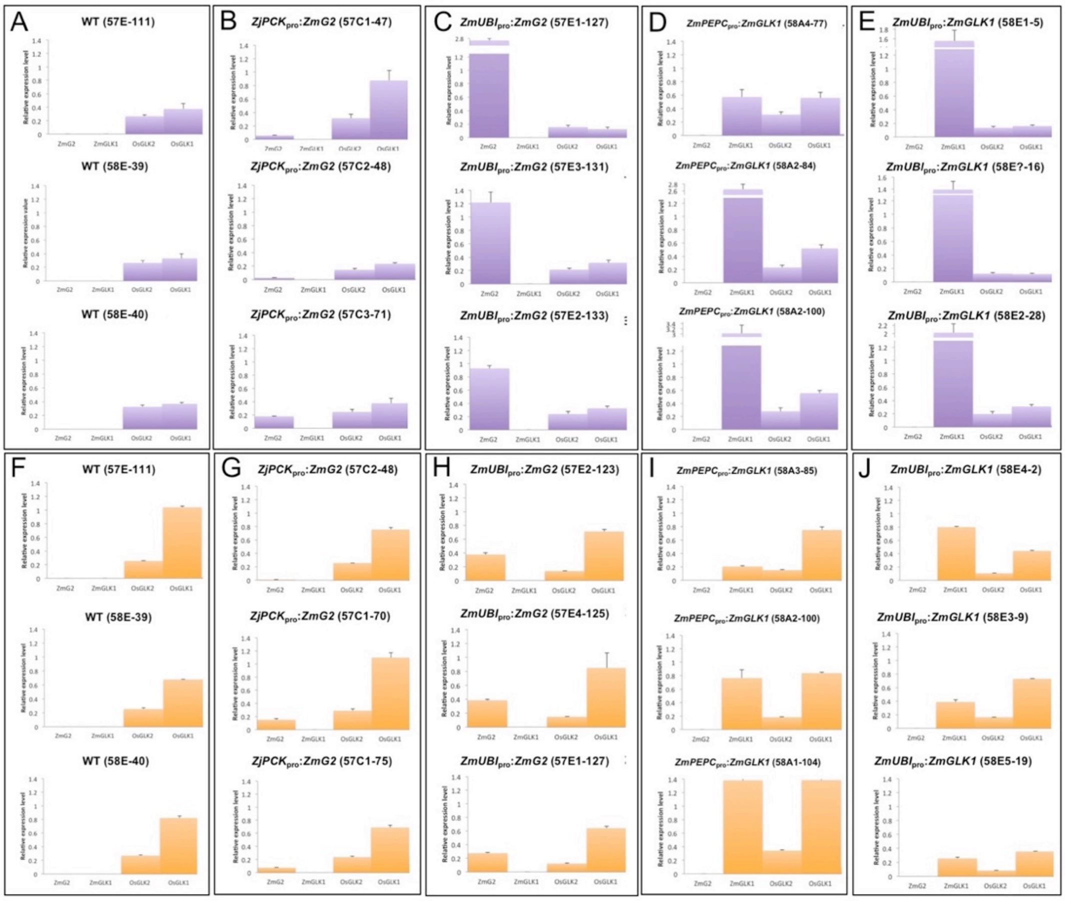

Figure S2. Relative transcript levels in *ZmG2* and *ZmGLK1* T1 transgenic lines. Related to Figures 2-5, Table 1.

A-J) Relative levels of *ZmG2* transgene, *ZmGLK1* transgene, *OsGLK2* and *OsGLK1* in 4th (A-E) and 7th (F-J) leaves of T1 Kitaake lines grown at 300 µmol photons m^{-2} s^{-1}. Transcript levels were measured by qRT-PCR and adjusted relative to actin transcript levels using the ΔCT value output from the StepOnePlus^{TM} system. Error bars indicate standard error computed from the mean of three experimental replicates. Transgene transcript levels were higher in leaf 4 (regardless of promoter) than leaf 7, whereas endogenous *OsGLK1* transcript levels were higher in leaf 7 than leaf 4. The bundle sheath preferential *ZjPCK*$_{pro}$ consistently drove the lowest levels of transgene expression. Transgene copy number in each line is shown in Figure S1. Lines are those used for analyses in Figures 2-5 and Table 1 of the main paper.

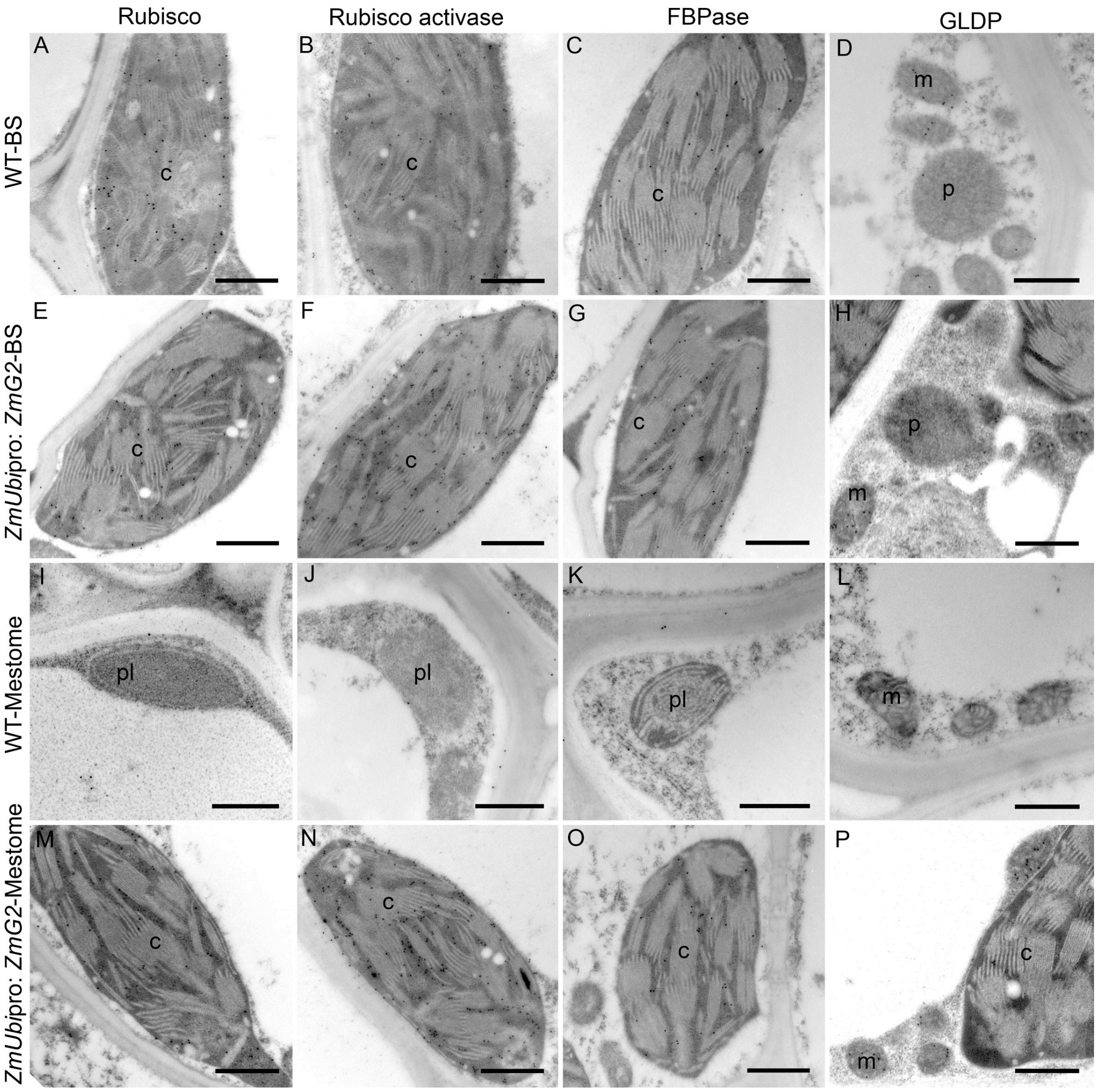

Figure S3. Immunolocalization of photosynthetic enzymes and mitochondrial glycine decarboxylase in vascular sheath cells of wild-type and transgenic lines. Related to Figure 3, Table 1.

A-P) Immuno-gold labeling of wild-type (WT) (A-D, I-L) and *ZmUBI*$_{pro}$*:ZmG2* transgenic Kitaake (E-H, M-P) bundle sheath (A-H) and mestome sheath (I-P) cells. Sections were reacted with antibodies against chloroplast localized RuBisCo (A, E, I, M), RuBisCo activase (B, F, J, N) and fructose-1,6-bis-phosphatase (FBPase) (C, G, K, O) or mitochondrial localized P subunit of glycine decarboxylase (GLDP) (D, H, L, P). All sections were prepared from leaf 7 of plants grown at 300 μmol photons m^{-2} s^{-1}. Scale bars = 500nm; c, chloroplast; m, mitochondria; p, peroxisome; pl, proplastid. See Table S1 for details of transgenic lines used, Figure S2 for transgene transcript levels. Quantification of RuBisco and RuBisco activase labeling is shown in Table 1 of main paper.

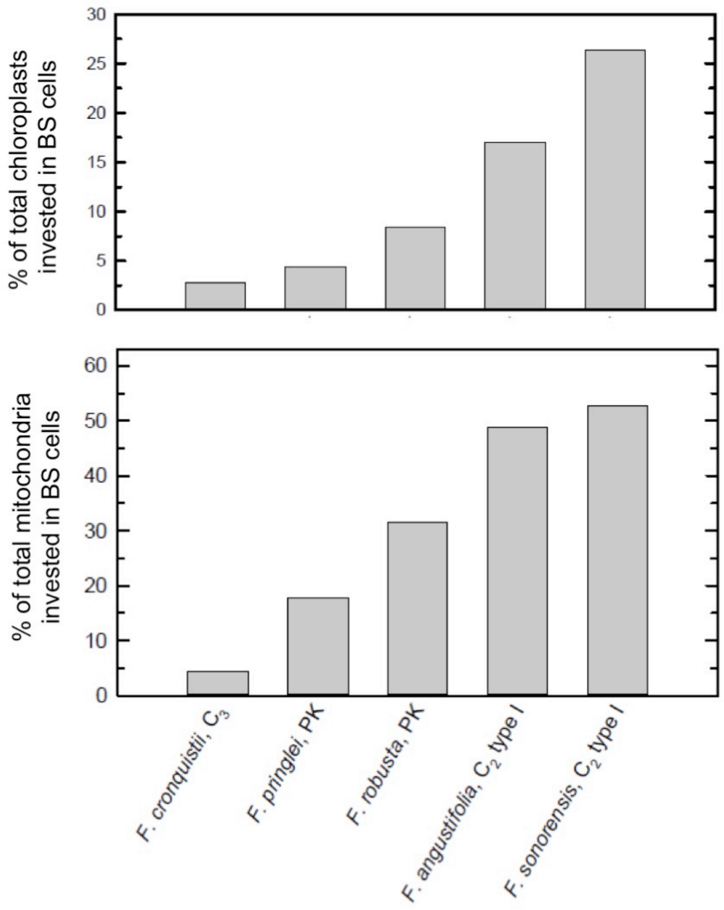

Figure S4. Relative investment of organelles in bundle sheath cells of *Flaveria* species. Related to Figure 5.

Percentage of total chloroplasts (upper panel) and mitochondria (lower panel) invested in bundle sheath cells (on a planar area basis) in the photosynthetic tissue of C_3, proto-Kranz (PK) and C_2 *Flaveria*. Organellar data are from [S1,S2] and the values for cell type % are extracted from M:BS ratios in Fig. 4 of [S3]. Calculations are as in experimental design section of methods and Table S5.

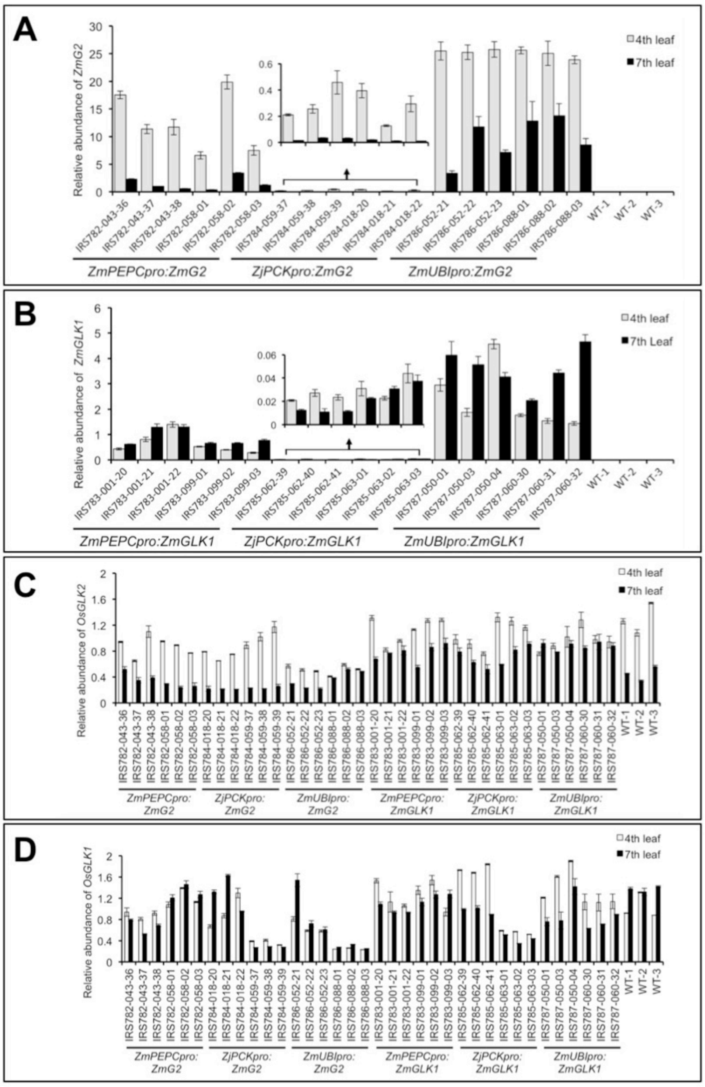

Figure S5. Transcript accumulation levels in *ZmG2* and *ZmGLK1* overexpression lines. Related to Figure 6.

A-D) Relative levels of *ZmG2* transgene (A), *ZmGLK1* transgene (B), *OsGLK2* (C) and *OsGLK1* (D) transcripts in 4th and 7th leaves of the IR64 lines genotyped in Figure S1. Transcript levels were measured by qRT-PCR and adjusted relative to actin transcript levels using the $2^{-\Delta CT}$ method. Error bars indicate standard error computed from the mean of three experimental replicates. As with equivalent Kitaake lines (Figure S2) *ZmG2* Transgene transcript levels were higher in leaf 4 (regardless of promoter) than leaf 7, however, *ZmGLK1* transcripts were similar at both developmental stages. A similar distinction was seen between the orthologous *OsGLK2* and *OsGLK1* transcripts, suggesting that sequence-specific post-transcriptional regulation may be operating. Lines are those used for analyses in Figure 6 of the main paper.

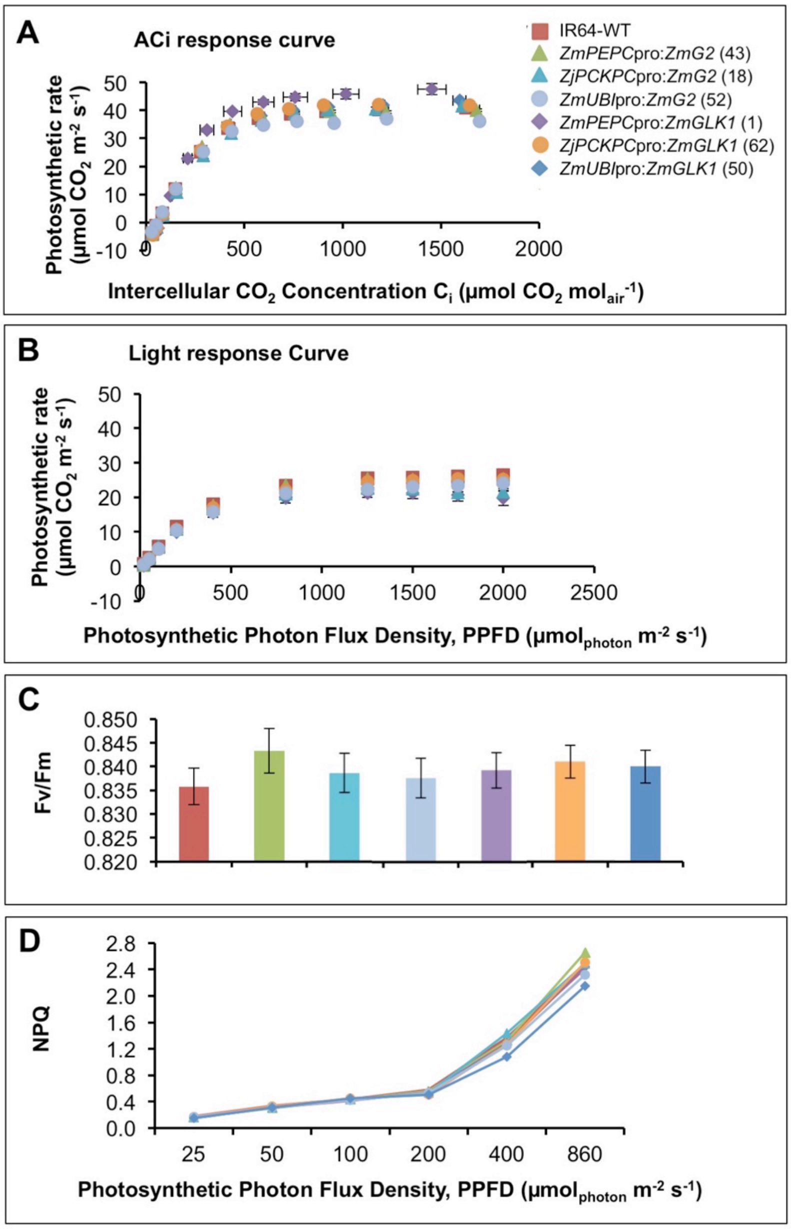

Figure S6. Overall photosynthetic parameters are not altered in rice lines expressing *ZmG2* or *ZmGLK1*. Related to Figure 6.

A) A-Ci response curve **B)** Light response curve **C)** Quantum yield (Fv/Fm) measurements **D)** Non-photochemical quenching (NPQ) response. Datapoints for each line are colour coded as in (A), with the line number indicated in parentheses. Error bars represent s.e.m. n= 9. For A & B three individuals were measured per line, with three experimental replicates in each case. For C & D, three different leaves were measured for each of three individuals per line. Transgene copy number and expression levels for each line are shown in Figure S1 and S5, respectively. Whole plant phenotypes and yield data for the same lines are shown in Figure 6 of the main paper.

	Cultivar	Original line number	# insertion sites	Leaf 4 single cell counts & qPCR	Leaf 7 single cell counts, TEM & qPCR
ZmPEPC$_{pro}$:ZmG2	IR64	IRS782-043	1		
ZmPEPC$_{pro}$:ZmG2	IR64	IRS782-058	1		
ZjPCK$_{pro}$:ZmG2	IR64	IRS784-018	1		
ZjPCK$_{pro}$:ZmG2	IR64	IRS784-059	1		
ZjPCK$_{pro}$:ZmG2	Kitaake	57C1	>3	C1-47	C1-70, 75
ZjPCK$_{pro}$:ZmG2	Kitaake	57C2	1	C2-48	C2-48
ZjPCK$_{pro}$:ZmG2	Kitaake	57C3	>2	C3-71	
ZmUBI$_{pro}$:ZmG2	IR64	IRS786-052	2		
ZmUBI$_{pro}$:ZmG2	IR64	IRS786-088	1		
ZmUBI$_{pro}$:ZmG2	Kitaake	57E1	1	E1-127	E1-127
ZmUBI$_{pro}$:ZmG2	Kitaake	57E2	1	E2-133	E2-123
ZmUBI$_{pro}$:ZmG2	Kitaake	57E3	1	E3-131	
ZmUBI$_{pro}$:ZmG2	Kitaake	57E4	2		E4-125
ZmPEPC$_{pro}$:ZmGLK1	IR64	IRS783-001	2		
ZmPEPC$_{pro}$:ZmGLK1	IR64	IRS783-099	2		
ZmPEPC$_{pro}$:ZmGLK1	Kitaake	58A1	2?		A1-104
ZmPEPC$_{pro}$:ZmGLK1	Kitaake	58A2	1	A2-84,100	A2-100
ZmPEPC$_{pro}$:ZmGLK1	Kitaake	58A3	1		A3-85
ZmPEPC$_{pro}$:ZmGLK1	Kitaake	58A4	1	A4-77	
ZjPCK$_{pro}$:ZmGLK1	IR64	IRS785-062	1		
ZjPCK$_{pro}$:ZmGLK1	IR64	IRS785-063	1		
ZmUBI$_{pro}$:ZmGLK1	IR64	IRS787-050	1		
ZmUBI$_{pro}$:ZmGLK1	IR64	IRS787-060	1		
ZmUBI$_{pro}$:ZmGLK1	Kitaake	58E1	1	E1-5	
ZmUBI$_{pro}$:ZmGLK1	Kitaake	58E2	1	E2-28	
ZmUBI$_{pro}$:ZmGLK1	Kitaake	58E3	1		E3-9
ZmUBI$_{pro}$:ZmGLK1	Kitaake	58E4	4		E4-2
ZmUBI$_{pro}$:ZmGLK1	Kitaake	58E5	1		E5-19
ZmUBI$_{pro}$:ZmGLK1	Kitaake	58E?	?	E-16	

Table S1. Summary of transgenic lines analyzed. Related to Figures 1-6.

Line	Planar Chloroplast Area (μm^2)	Chloroplast Number/ BS Cell	Chloroplast Number/BS Cell Volume ($\mu m^{-3} \times 10^{-3}$)	BS Cell Volume, (μm^3)
**Leaf 4**				
WT	10.58 ± 0.37^a	14 (6-21)	2.90 ± 0.27^a	5518 ± 479^a
ZjPCK$_{pro}$:*ZmG2* (3)	11.76 ± 0.35^a	15 (7-26)	2.84 ± 0.14^a	6386 ± 539^a
ZmUBI$_{pro}$:ZmG2 (3)	15.85 ± 0.69^b	13 (8-21)	3.44 ± 0.27^a	5088 ± 462^a
ZmPEPC$_{pro}$:*ZmGLK1* (2)	11.68 ± 0.36^a	15(10-20)	4.03 ± 0.31^a	5339 ± 621^a
ZmUBI$_{pro}$:*ZmGLK1* (2)	13.67 ± 0.39^b	13 (9-19)	3.08 ± 0.22^a	5076 ± 335^a
**Leaf 7**				
WT	8.82 ± 0.28^a	12 (9-19)	3.26 ± 0.29^a	4803 ± 320^a
ZjPCK$_{pro}$:*ZmG2* (2)	10.94 ± 0.32^b	15 (11-22)	3.63 ± 0.23^a	4720 ± 206^a
ZmUBI$_{pro}$:*ZmG2* (3)	15.55 ± 0.50^c	15(11-21)	4.05 ± 0.30^a	4606 ± 324^a
ZmPEPC$_{pro}$:*ZmGLK1* (3)	12.80 ± 0.38^d	14 (11-20)	3.77 ± 0.23^a	4541 ± 322^a
ZmUBI$_{pro}$:*ZmGLK1* (3)	13.30 ± 0.57^d	15 (9-22)	3.89 ± 0.27^a	4427 ± 280^a

Table S2. Quantification of chloroplast size and number in bundle sheath cells. Related to Figure 2.

Planar chloroplast area, number, and number per cell volume in isolated bundle sheath (BS) cells from leaves 4 and 7 of T1 transgenic Kitaake and wild-type (WT) lines grown at 300 µmol photons $m^{-2} s^{-1}$. Transgene transcript levels in each leaf at the time of harvesting are shown in Figure S2. Mean ± s.e.m. (except chloroplast number which is represented by median and range), n = 45 i.e. 15 BS cells from each of three individuals representing at least 2 independent transgenic lines (number of independent lines in each sample is indicated in parentheses after construct name). Statistically distinct groups for each leaf are indicated by superscripted letters ($p<0.05$) as determined by a Games-Howell test of variance. Data correspond to Figure 2 in main text.

	WT	*ZjPCK*$_{pro}$: *ZmG2*	*ZmUBI*$_{pro}$: *ZmG2*	*ZmPEPC*$_{pro}$: *ZmGLK1*	*ZmUBI*$_{pro}$: *ZmGLK1*
**Planar chloroplast area/ planar cell area (%)**					
BS	5.3±0.7^{a}	7.2±0.7^{ab}	12.9±0.7^{c}	9.3±0.7^{b}	7.0±0.7^{ab}
MS	0.4±0.2^{a}	1.0±0.6^{ab}	16.3±1.9^{c}	1.3±0.3^{b}	7.2±1.3^{d}
M*	50.9±1.5^{a}	50.3±1.8^{a}	54.6±1.2^{a}	48.7±1.8^{a}	48.4±2.1^{a}
**Planar mitochondria area/ planar cell area (%)**					
BS	0.5±0.2^{a}	0.4±0.2^{a}	2.9±0.2^{b}	0.5±0.2^{a}	1.9±0.2^{b}
MS	1.4±0.2^{a}	1.8±0.2^{ab}	2.3±0.2^{b}	1.5±0.2^{ab}	2.0±0.2^{ab}
M	2.0±0.1^{a}	3.3±0.3^{b}	2.3±0.2^{ab}	1.3±0.1^{c}	1.9±0.1^{a}
**Planar mitochondria #/ planar cell area, μm^{-2} ×10^{-3}**					
BS	34.0±3.9^{ab}	26.1±3.9^{a}	44.5±3.9^{b}	48.4±3.9^{b}	29.3±3.9^{a}
MS	107.5±18.4^{a}	103.7±15.3^{a}	136.6±12.1^{a}	118.5±14.3^{a}	154.4±14.1^{a}
M	57.4±3.8^{a}	70.9±4.2^{a}	63.1±4.3^{a}	63.4±5.0^{a}	55.8±4.2^{a}

Table S3. Quantification of relationship between chloroplast and mitochondrial size in leaf 7 of wild-type and transgenic lines. Related to Figures 3 and 4, Table 1.

Planar chloroplast and mitochondria area/planar cell area of bundle sheath (BS), mestome sheath (MS) and mesophyll (M) cells of leaf 7 from transgenic Kitaake and wild-type (WT) plants grown at 300 μmol photons m^{-2} s^{-1}. Transgene transcript levels in each leaf at the time of harvesting are shown in Figure S2. Cell and organelle areas were calculated from TEM images; values are mean ± s.e.m; n=45 i.e. 15 cells/individual, 3 individuals/construct. Individuals represent at least two independent transgenic events for each construct (see Table S1 for line numbers, and Figure S1 for transgene copy number in each line). Statistically distinct groups are indicated by superscripted letter within each row ($p<0.05$) by a Tukey's test of variance for normally distributed data (asterisk) and a Games-Howell test of variance for the rest of the data. Data correspond to Figures 3 & 4 and Table 1 in main text.

	WT	*ZjPCK*$_{pro}$: *ZmG2*	*ZmUBI*$_{pro}$: *ZmG2*	*ZmPEPC*$_{pro}$: *ZmGLK1*	*ZmUBI*$_{pro}$: *ZmGLK1*
BS-M	35.6±11.1	51.1±8.0	64.4±5.9*	60.0±6.7*	56.7±3.3*
BS-MS	24.4±2.2	33.3±3.8	44.4±11.8*	42.2±9.7*	28.9±2.2

Table S4. Relative frequency of plasmodesmata between cell-types in wild-type and transgenic lines. Related to Figure 4.
Percentage of bundle sheath cells that had plasmodesmata in walls shared with adjacent mesophyll (BS-M) or mestome sheath cells (BS-MS) in leaf 7 of transgenic Kitaake and wild-type (WT) plants grown at 300 μmol photons $m^{-2} s^{-1}$. Values were calculated from TEM images and are mean ± s.e.m; n = 45 i.e. 15 cells/individual, 3 individuals/construct. Individuals represent at least two independent transgenic events for each construct (see Table S1 for line numbers, Figure S1 for transgene copy number in each line and Figure S2 for transgene transcript levels). Asterisks indicate lines with frequency of plasmodesmata significantly higher than WT, as measured by a General Linear Model.

Species	Mesophyll: Vascular Sheath area ratio	Area per BS cells, μm^2	Chloroplast area/ mesophyll cell area, %	Chloroplast area/ vascular sheath cell area, %	Mitochondria area/ mesophyll cell area, %	Mitochondria area/ vascular sheath cell area, %	Mesophyll tissue area/ total photosynthetic tissue area, %	Vascular sheath tissue area/ total photosynthetic tissue area, %	Photosynthetic tissue chloroplast in mesophyll, %	Photosynthetic tissue chloroplast in vascular sheath, %	Photosynthetic tissue mitochondria in mesophyll, %	Photosynthetic tissue mitochondria in vascular sheath, %
Oryza sativa(WT)	7.1±0.7	90±5	50.9±1.5	BS: 5.3±0.7<br>MS: 0.4±0.2	2.0±0.1	BS: 0.5±0.2<br>MS: 1.4±0.2	85.5±2.5	14.5±2.5	98.3±0.2	1.7±0.2	93.3±0.6	6.7±0.6
Oryza sativa (*ZjPCK*$_{pro}$:ZmG2)	4.7±0.5	123±7	50.3±1.8	BS:7.2±0.7<br>MS: 1.0±0.6	3.3±0.3	BS: 0.4±0.2<br>MS: 1.8±0.2	82.4±1.1	17.6±1.1	96.9±0.5	3.1±0.5	93.7±0.8	6.3±0.8
Oryza sativa (ZmUBI$_{pro}$*:ZmG2)*	4.6±0.3	102±7	54.6±1.2	BS: 12.9±0.7<br>MS: 16.3±1.9	2.3±0.2	BS: 2.9±0.2<br>MS: 2.3±0.2	81.8±0.8	18.2±0.8	94.8±1.1	5.5±1.1	79.2±4.3	20.8±4.3
Oryza sativa (*ZmPEPC*$_{pro}$:ZmGLK1)	5.4±0.6	103±5	48.7±1.8	BS:9.3±0.7<br>MS: 1.3±0.3	1.3±0.1	BS: 0.5±0.2<br>MS: 1.5±0.2	83.8±0.6	16.2±0.6	95.8±1.1	3.5±0.9	87.8±1.1	12.2±1.1
Oryza sativa (*ZmUBI*$_{pro}$*:ZmGLK1*)	7.2±2.9	122±7	48.4±2.1	BS:7.0±0.7<br>MS: 7.2±1.3	1.9±0.1	BS: 1.9±0.2<br>MS: 2.0±0.2	82.9±0.7	17.3±2.2	96.9±0.7	4.3±1.1	78.4±6.0	21.6±6.0
Dicanthelium oligosanthes, C_3PACMAD	5.7±0.5	214±20	26.9±1.8	3.8±0.4	0.28±0.03	0.50±0.05	84.8±0.9	15.2±0.9	97.5±0.1	2.5±0.1	90.5±1.9	9.5±1.9
Steinchisma laxum, PK	2.4±0.4	582±107	30.3±1.6	4.9±0.5	0.27±0.02	0.39±0.03	67.6±4.4	32.4±4.4	93.0±1.2	6.8±1.2	76.1±0.8	23.9±0.8
S. hians, C_2	2.2±0.1	402±30	28.2±1.6	11.2±0.8	0.81±0.06	0.39±0.04	68.1±1.8	31.9±1.8	84.0±2.9	16.0±2.9	51.1±3.4	48.9±3.4
Homolepis aturensis, C_2	2.1±0.2	734±48	17.7±1.2	8.3±0.7	0.73±0.06	0. 30±0.06	66.4±1.6	33.6±1.6	80.8±2.8	19.2±2.8	44.2±2.9	55.8±2.9
Panicum virgatum, C_4 NAD-ME	1.8±0.1	509±39	12.3±0.9	14.6±1.1	0.74±0.07	0.09±0.02	63.2±2.1	36.8±2.1	53.6±3.0	46.4±3.0	17.2±1.4	82.8±1.4
Setaria viridis, C_4 NADP-ME	4.7±0.4	204±15	18.5±1.5	41.8±1.7	0.64±0.08	0.13±0.02	82.0±1.1	18.0±1.1	61.4±1.5	38.6±1.5	47.2±5.4	52.8±5.4
Flaveria cronquistii, C_3	17	892	6.6±0.9	3.1±0.2	0.5±0.1	0.4±0.1	94.4	5.6	97.2	2.8	95.5	4.5
F. pringlei, PK	8.4	746	9.9±1.5	3.6±0.4	0.5±0.1	0.9±0.1	89.4	10.6	95.6	4.4	82.4	17.6
F. robusta, PK	6.5	437	11.7±2.1	6.9±3.3	0.4±0.1	1.2±0.1	86.7	13.3	91.5	8.5	68.4	31.6
F. sonorensis, C_2 type I	2.7	965	11.6±1.0	11.6±1.0	0.4±0.1	1.2±0.3	73	27	73.6	26.4	47.4	52.6
F. angustifolia, C_2 type I	4.4	524	11.0±1.4	10.2±0.8	0.5±0.1	2.1±0.2	81.5	18.5	83	17	51.2	48.8
F. anomala, C_2 type II	3.3	801	16.4±0.2	13.6±0.6	NA	NA	76.7	23.3	79.9	20.1	NA	NA
F. linearis, C_2	3.5	1138	10.2±1.4	13.2±0.1	NA	NA	77.8	22.2	73	27	NA	NA
F. floridana, C_2 type II	3.8	826	7.9±0.4	11.5±0.2	NA	NA	79.2	20.8	72.3	27.7	NA	NA
F. ramosissima, C_2 type II	2.7	947	9.0±2.8	13.4±1.3	NA	NA	73	27	64.5	35.5	NA	NA
F. brownii, C_4-like	1.4	855	7.4±1.5	17.1±1.8	NA	NA	58.3	41.7	37.7	62.3	NA	NA
F. vaginata, C_4-like	1.3	613	14.6±1.3	36.5±1.0	NA	NA	56.5	43.5	34	66	NA	NA
F. bisentis, C_4	1.5	667	13.7±1.0	31.0±1.4	NA	NA	60	40	39.9	60.1	NA	NA
F. trinervia, C_4	2.2	538	15.0±0.5	36.7±3.4	NA	NA	68.8	31.3	49.3	50.7	NA	NA

Table S5. Percentage of chloroplasts and mitochondria invested in each cell-type in wild-type rice, *GLK* transgenic lines, various PACMAD grasses and *Flaveria*. Related to Figure 5.

The % of organelles invested in each cell-type is quantified as the proportion of organellar area in that cell-type, as compared to the total organellar area across all photosynthetic tissues (see experimental design section of methods for full details). The total photosynthetic tissue area is defined as the sum of the planar leaf area covered by both mesophyll and vascular sheath cells. The total organellar area in all photosynthetic tissue is quantified as the sum of the fractional organelle area in each cell-type. Mean ± s.e.m., n = 3 for rice and each PACMAD grass. *Dichanthelium*, *Steinchisma* and *Homolepis* data are from [S4] *Flaveria* organellar data are from [S1,S2] and the values for cell type % in *Flaveria* species are extracted from M:BS ratios in Fig. 4 of [S3]. Data correspond to Figure 5 in main text and Figure S4.

Primer name	Sequence (5' to 3')
ZmG2-cloningF	GGGGACAAGTTTGTACAAAAAAGCAGGCTATGCTTGAGGTGTCGACGCTG
ZmG2-cloningR	GGGGACCACTTTGTACAAGAAAGCTGGGTTAGTATGTCATCCGGTGGCGC
ZmGLK1-cloningF	GGGGACAAGTTTGTACAAAAAAGCAGGCTATGCTTGCAGTGTCGCCGTC
ZmGLK1-cloningR	GGGGACCACTTTGTACAAGAAAGCTGGGTTCATCCACAAGCTTGGGCAC
pVec8F (*UBI*pro)	TTTAGCCCTGCCTTCATACG
pVec8R (*nos*T)	ATTGCCAAATGTTTGAACGA
pSC110F(*PEPC*pro)	ACGACTCCCCATCCCTATTT
pSC210F(*PCK*pro)	CTGCTGCTGCTGCTCTCTC
pSC1/2/310-R	AAGACCGGCAACAGGATTC
HPTFpr3	AAACTGTGATGGACGACACC
HPTRpr2	CTATCAGAGCTTGGTTGACG
ZmG2-F	CATGGTGGACGACAACCTC
ZmG2-R	CACATGTTTGCTCCAACGAC
ZmGLK1-F	GGACCTGGATTTCGACTTCA
ZmGLK1-R	CACTCCCCTTTCCCTTCTTC
Actin-F	GGCACCACACCTTCTACAAT
Actin-R	CTCACACCATCACCAGAGT
OsGLK1-F	AGCTGCGAGATTTCCTGCTC
OsGLK1-R	ATAGCTGCGTCGATGCTCTC
OsGLK2-F	AGGGGAGAGATTTTGGGATGC
OsGLK2-R	TTCCTTCACGTCTTCCTTGG

Table S6. PCR primers used. Related to STAR Methods.

SUPPLEMENTAL REFERENCES

S1. Stata, M., Sage, T.L., Hoffmann, N., Covshoff, S., Ka-Shu Wong, G., and Sage, R.F. (2016). Mesophyll chloroplast investment in C_3, C_4 and C_2 species of the genus *Flaveria*. Plant Cell Physiol. *57*, 904–918.

S2. Sage, T.L., Busch, F.A., Johnson, D.C., Friesen, P.C., Stinson, C.R., Stata, M., Sultmanis, S., Rahman, B.A., Rawsthorne, S., and Sage, R.F. (2013). Initial events during the evolution of C_4 photosynthesis in C_3 species of *Flaveria*. Plant Physiol. *163*, 1266–1276.

S3. McKown, A.D., and Dengler, N.G. (2007). Key innovations in the evolution of Kranz anatomy and C_4 vein pattern in *Flaveria* (Asteraceae). Am. J. Bot. *94*, 382–399.

S4. Khoshravesh, R., Stinson, C.R., Stata, M., Busch, F.A., Sage, R.F., Ludwig, M., and Sage, T.L. (2016). C_3–C_4 intermediacy in grasses: organelle enrichment and distribution, glycine decarboxylase expression, and the rise of C_2 photosynthesis. J. Exp. Bot. *67*, 3065–3078.
